# Vanadium Substitution Dictates H Atom Uptake at Lindqvist-type Polyoxotungstates

**DOI:** 10.1021/acs.inorgchem.4c03873

**Published:** 2024-11-20

**Authors:** Dominic Shiels, Zhou Lu, Magda Pascual-Borràs, Nathalia Cajiao, Thompson V. Marinho, William W. Brennessel, Michael L. Neidig, R. John Errington, Ellen M. Matson

**Affiliations:** † Department of Chemistry, University of Rochester, Rochester, New York 14627, United States; ‡ NUPOM Lab, Chemistry, School of Natural & Environmental Sciences, Newcastle University, Newcastle upon Tyne NE1 7RU, U.K.; § Inorganic Chemistry Laboratory, Department of Chemistry, University of Oxford, South Parks Road, Oxford OX1 3QR, U.K.

## Abstract

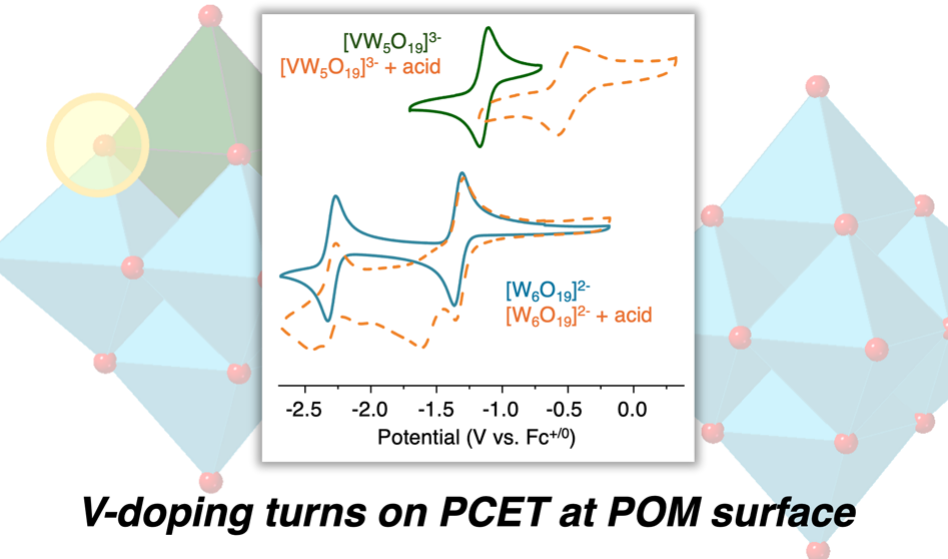

Understanding how modification of molecular structures changes the thermochemistry of H atom uptake can provide design criteria for the formation of highly active catalysts for reductive transformations. Herein, we describe the effect of doping an atomically precise polyoxotungstate with vanadium on proton-coupled electron transfer (PCET) reactivity. The Lindqvist-type polyoxotungstate [W_6_O_19_]^2–^ displays reversible redox chemistry, which was found to be unchanged in the presence of acid, indicating an inability to couple reduction with protonation. However, the incorporation of a single vanadium center into the structure significantly changes the reactivity, and the potential required for one-electron reduction of [VW_5_O_19_]^3–^ was shown to vary with the strength of the acid added. Construction of a potential-p*K*_a_ diagram allowed assessment of the thermodynamics of H atom uptake, indicating BDFE(O–H) ≈ 64 kcal/mol, while chemical synthesis of the reduced/protonated derivative (TBA)_3_[VW_5_O_19_H] was used to probe the position of protonation.

## Abstract 2

Electrochemical studies show that the potential required for the one-electron reduction of [VW_5_O_19_]^3−^ changes in the presence of acid. Performing the same experiments on [W_6_O_19_]^2−^ reveals that the redox chemistry of this polyoxometalate is acid-independent. This indicates that doping a Lindqvist-type polyoxotungstate with vanadium “switches on” proton-coupled electron transfer (PCET) reactivity. Performing experiments with a range of acids allows extraction of a BDFE(O−H) value of ≈64 kcal/mol^−1^ for the reduced/protonated compound [VW_5_O_18_(OH)]^3−^.

## Introduction

Polyoxotungstates are a class of polyoxometalate (POM) that consist of multiple redox-active tungsten atoms linked with bridging oxide moieties, resulting in the formation of discrete, three-dimensional assemblies.^1^ The peripheral morphology of polyoxotungstates resembles the surface of metal oxide structures, with alternating terminal and bridging oxygen atoms. Polyoxotungstates exhibit stability and solubility in organic and aqueous media, rendering them amenable to atomically precise characterization using molecular analytical techniques. These traits make polyoxotungstates intriguing models for the surface reactivity of redox-active metal oxide materials.^2−5^

One of the most advantageous and well-studied properties of polyoxotungstates is their ability to undergo reversible redox reactions. The modular character of these molecular metal oxide compounds allows for extensive tuning of both the number of redox events, as well as the electrochemical potential at which electrons are transferred.^6−11^ With most relevance to the work presented here, heterometal substitution can profoundly change the electronic structure of a polyoxotungstate framework, which in turn dictates the redox chemistry. Replacement of one of more “W=O” units with heterometallic “{M-X}^n+^” units can lead to significant changes in the overall charge of the polyoxotungstate, which influences the redox potentials of native W(V)/W(VI) redox couples. Furthermore, incorporation of redox-active metals can serve to add additional, heterometal-specific redox events to the overall redox manifold. For example, installation of vanadium (which is the focus of this work) sites into the [PW_12_O_40_]^3–^ has already been shown to provide access to additional V(IV)/V(V) redox events (with the number depending on the number of V dopants) alongside the intrinsic tungsten-based redox chemistry of the Keggin anion.^12,13^

In addition to structural modifications (i.e., heterometal substitution) that influence the physicochemical properties of polyoxotungstates, the electrochemistry of these metal oxide assemblies has been shown to be sensitive to the concentration and p*K*_a_ of organic acids.^14−17^ In the absence of protons, most polyoxotungstates exhibit multiple single-electron redox events. Addition of acid results in anodic shifts; in some cases, individual redox events merge to form multielectron-multiproton processes. This phenomenon suggests that these POMs, in their reduced forms, possess reactive hydrogen equivalents at their surfaces. Recently our research team has published a report describing the thermochemistry and kinetics of H atom uptake and transfer at the surface of homometallic polyoxotungstates.^18^ Initial studies focused on the plenary Keggin-type phosphotungstate [PW_12_O_40_]^3–^, demonstrating that four H atom equivalents can be stored at the surface of the cluster, with BDFE(O–H)_avg_ values of ∼45 kcal mol^–1^. The thermochemistry of the reactive O–H moieties formed upon reduction of the assembly in the presence of a sufficiently strong organic acid (p*K*_a_ < 7) explains the previously observed evolution of H_2_ from protonated and reduced versions of the POM.^18^

**Figure 1:**
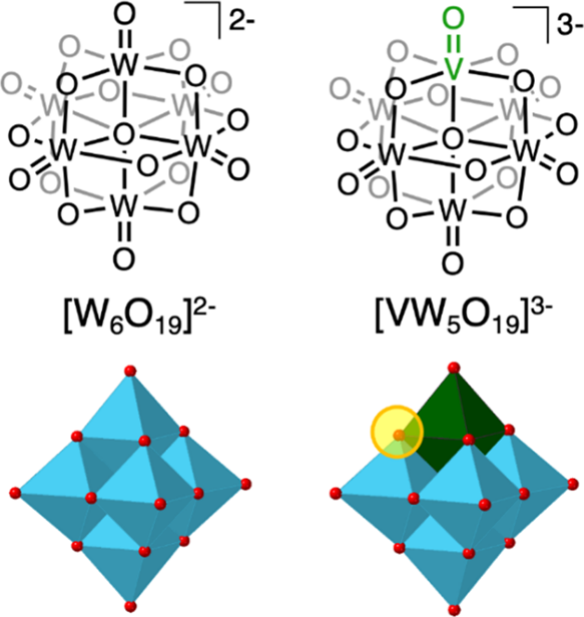
Homo- (left) and hetero(right) metallic polyoxotungstates, [MW_5_O_19_]^n–^ (M = W, *n* = 2; M = V, *n* = 3), studied in this work. Yellow circle indicates site of H atom uptake at the vanadium-substituted polyoxotungstate.

Given that the redox chemistry of polyoxotungstates has been demonstrated to be highly sensitive to heterometal substitution,^12,13^ our interests have turned to understanding the impact of dopants on the thermochemistry of H atom uptake and transfer at the surface of polyoxotungstates. Our group’s previous works have extensively demonstrated the ability of vanadium-based polyoxo-alkoxides to undergo proton-coupled electron transfer (PCET).^19−25^ Considering this, it was postulated that doping a Lindqvist-type polyoxotungstate with vanadium could influence the thermodynamics of H atom uptake around the dopant site (i.e., V–O–W bridging sites). Thus, we now report an extension of our studies to a vanadium-substituted, Lindqvist-type polyoxotungstate assembly, **[VW**_**5**_**O**_**19**_**]**^**3–**^ (Figure 1). Our results reveal that the presence of a heterometal has a profound impact on the thermochemistry of H atom uptake; the vanadium-doped polyoxotungstate assembly has a high affinity for H atom equivalents, whereas the all-tungsten derivative is unreactive. These differences in reactivity can be attributed to the change in charge upon doping the polyoxotungstate framework, and differences in the location of reduction of the Lindqvist-type assembly as a function of metal composition. These effects are synergistic and combine to increase the surface basicity of the framework which is likely a key factor in making H atom uptake more thermodynamically favorable for **[VW**_**5**_**O**_**19**_**]**^**3–**^ than for either **[W**_**6**_**O**_**19**_**]**^**2–**^ (discussed here) or [PW_12_O_40_]^3–^ (previously reported).^18^ The overall consequence of this is that PCET reactivity for Lindqvist-type polyoxotungstates can be “switched on” by doping the framework with vanadium.

## Experimental Section

### General Considerations

All air- and moisture-sensitive manipulations were carried out using a standard high vacuum line and Schlenk techniques, or in an MBraun inert atmosphere drybox containing an atmosphere of purified dinitrogen. All solids were dried under high vacuum in order to be brought into the glovebox. Solvents for air- and moisture-sensitive manipulations were dried and deoxygenated using a Glass Contour Solvent Purification System (Pure Process Technology, LLC) and stored over activated 4 Å molecular sieves (Fisher Scientific) prior to use. Deuterated solvents for NMR spectroscopy were purchased from Cambridge Isotope Laboratories and, after three freeze–pump–thaw cycles, were stored in the glovebox over activated 3 Å molecular sieves. (TBA)_2_[W_6_O_19_] and (TBA)_3_[VW_5_O_19_] were synthesized according to literature methods.^26,27^ All the remaining chemicals were purchased from commercial sources (Fisher Scientific, VWR, and Sigma-Aldrich) and used without further purification.

### Physical Measurements

^1^H and ^51^V NMR spectra were recorded at room temperature on a 400 MHz Bruker AVANCE spectrometer, a 500 MHz Bruker AVANCE spectrometer, or a 500 MHz JEOL JNM-ECZR spectrometer locked on the signal of deuterated solvents. All chemical shifts are reported relative to the chosen deuterated solvent as a standard. Electronic absorption measurements were recorded at room temperature in anhydrous MeCN or DCM in sealed 1 cm quartz cuvettes using an Agilent Cary 6000i UV–vis–NIR spectrophotometer. Infrared spectroscopy was conducted with a PerkinElmer Spectrum 3 FT-IR Spectrometer. X-band electron paramagnetic resonance (EPR) measurements were carried out on a Bruker EMXplus spectrometer (microwave frequency of 9.382 GHz) at 10 K. The EPR spectra were simulated using Easy-Spin version 6.0.2.^28^ Elemental analysis data were obtained from the Elemental Analysis Facility at the University of Rochester. Microanalysis samples were weighed with a PerkinElmer model AD6000 autobalance, and their compositions were determined with a PerkinElmer 2400 series II analyzer. Air-sensitive samples were handled in a VAC Atmospheres glovebox.

Cyclic voltammetry (CV) experiments were recorded with a Bio-Logic SP-150 potentiostat/galvanostat and the EC-Lab software suite. All measurements were performed in a three-electrode system cell configuration that consisted of a glassy-carbon (ø = 3.0 mm) as working electrode (CH Instruments), a Pt wire as the counter electrode (CH Instruments), and an Ag/Ag^+^ nonaqueous reference electrode with 0.01 M AgNO_3_ in 0.05 M TBA(PF_6_) in acetonitrile (BASi). The supporting electrolyte, tetrabutylammonium hexafluorophosphate TBA(PF_6_) was purchased from Sigma-Aldrich, recrystallized three times using hot ethanol, and stored under dynamic vacuum for a minimum of 2 days prior to use. All electrochemical measurements were performed at room temperature (∼22 °C) in a nitrogen-filled, UniLab MBraun inert atmosphere drybox. All glassware for CV experiments was dried for a minimum of 4 h and cooled in an evacuated antechamber prior to use in the drybox. Acetonitrile was dried and deoxygenated on a Glass Contour System (Pure Process Technology, LLC) and stored over 3 Å molecular sieves purchased from Fisher Scientific and activated prior to use.

Bulk electrolysis experiments were performed in a H-cell with a glass frit separator (porosity = 10–16 μm, Pine Research) using a Bio-Logic SP 150 potentiostat/galvanostat. In all experiments, an active species concentration of 1 mM was used. The working electrode compartment contained 10 mL of the active species with 0.1 M supporting electrolyte TBA(PF_6_) in MeCN, while the counter electrode compartment contained 10 mL of 0.1 M supporting electrolyte in the same solvent (MeCN). A Pt mesh working electrode and a Pt wire counter electrode were used. Bulk electrolysis experiments were carried out using the chronoamperometry techniques available in EC-Lab software suite at constant potentials, selected from CV.

To assess the thermochemistry of proton-coupled electron transfer at the surface of the M-doped polyoxotungstate clusters, potential-p*K*_a_ diagrams were constructed following the method reported by Dempsey et al.^29^ The specifics of the experiments described here are as follows: a 5 mL sample was prepared in acetonitrile containing 1 mM of the desired cluster (**(TBA)**_**2**_**[W**_**6**_**O**_**19**_**]** or **(TBA)**_**3**_**[VW**_**5**_**O**_**19**_**]**) and 0.1 M TBA(PF_6_) as a supporting electrolyte. An initial cyclic voltammogram was collected to ensure purity of the sample. An aliquot of a stock solution containing the desired organic acid was added to the sample and allowed to stir for 5 min, whereupon a cyclic voltammogram was collected. Organic acids used in this manuscript were generated following a procedure published previously by our laboratory.^21^ At completion of each experiment, a sample of ferrocene was added and an additional cyclic voltammogram was collected in order to properly reference to the Fc^+/0^ couple.

### X-ray Crystallography

Single crystals were placed onto a nylon loop and mounted on a Rigaku XtaLAB Synergy-S Dualflex diffractometer equipped with a HyPix-6000HE HPC area detector for data collection at 100.00(10) K. A preliminary set of cell constants and an orientation matrix were calculated from a small sampling of reflections.^30^ A short pre-experiment was run, from which an optimal data collection strategy was determined. The full data collection was carried out using a PhotonJet (Cu) X-ray source. After the intensity data were corrected for absorption, the final cell constants were calculated from the xyz centroids of the strong reflections from the actual data collection after integration.^30^ The structure was solved using SHELXT and refined using SHELXL.^31,32^ Most or all non-hydrogen atoms were assigned from the solution. Full-matrix least-squares/difference Fourier cycles were performed which located any remaining non-hydrogen atoms. All non-hydrogen atoms were refined with anisotropic displacement parameters. All hydrogen atoms were placed in ideal positions and refined as riding atoms with relative isotropic displacement parameters.

### Computational Details

All calculations were performed using Gaussian 09 package.^33^ All structures were optimized using B3LYP hybrid functional.^34−36^ The LANL2DZ(f) effective core potential basis set was applied for W and V while 6-31G(d,p) pople basis set was used for the remaining atoms.^37−40^ Solvent effects were included via implicit continuum model (IEF-PCM)^41^ to simulate the effects of acetonitrile. The nature of all the stationary points were verified with frequencies calculations. All the energies reported correspond to the computed Gibbs energies in solution, electrochemical steps are reported either in V or eV, and chemical steps in eV. All reported potentials are referenced to Fc/Fc^+^ couple in acetonitrile.^42^ For the energy of the proton the energy reported by Truhlar et al. was employed.^43^ Calculated p*K*_a_ values were obtained from the free energy of deprotonation steps using the standard equation:^44^
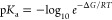


#### Synthesis of (TBA)_4_**[VW**_**5**_**O**_**19**_**]** (**[VW**_**5**_**O**_**19**_**]**^**4–**^)

In a Schlenk flask (or airtight screw top pressure vessel), (TBA)_3_[VW_5_O_19_] (1 g, 0.5 mmol, 1 equiv) was suspended in MeCN (10 mL). An excess of Zn (33 mg, 2.5 mmol, 5 equiv) and a stoichiometric amount of TBA(Cl) (140 mg, 0.5 mmol, 1 equiv) was added. The mixture was heated at 70 °C under an inert atmosphere for 16 h. During this time the remaining (TBA)_3_[VW_5_O_19_] dissolved, and a dark red/purple solution formed. The solution was allowed to cool to room temperature before the mixture was filtered through a bed of Celite. The solvent was then removed under reduced pressure and the residue was triturated with THF (3 × 5 mL) and Et_2_O (3 × 5 mL). A free-flowing red/purple powder is obtained (0.92 g, 82% yield). The crude material can be recrystallized by vapor diffusion of Et_2_O into a saturated solution of the crude material dissolved in MeCN. ^1^H NMR (500 MHz, CD_3_CN) δ 0.96 (s, 48 H), 1.39 (s, 32 H), 1.64 (s, 32 H), 3.20 (s, 32 H). ^51^V NMR (500 MHz, CD_3_CN) No signal, oxidized [VW_5_O_19_]^3–^ appears at −508 ppm. λ_max_ (MeCN) = 412 nm (ε_max_ = 420 M^–1^ cm^–1^), 510 nm (ε_max_ = 240 M^–1^ cm^–1^), and 984 nm (ε_max_ = 520 M^–1^ cm^–1^). FTIR, ν_max_/cm^–1^: 2959, 2934, 2873, 1666(w), 1479(s), 1381, 1152(w), 1107(w), 1056(w), 1027(w), 990, 933(s), 883, 787(s), 638, 572, 554. Formula: (TBA)_4_[VW_5_O_19_].THF, Anal. Calcd for C_68_H_152_N_4_VW_5_O_20_ (mol. wt. 2316.117 g/mol): C, 35.26%; H, 6.62%; N, 2.42%. Found: C, 35.07%; H, 6.68%; N, 2.46%.

#### Synthesis of (TBA)_3_**[VW**_**5**_**(OH)O**_**18**_**]** (**[V(OH)W**_**5**_**O**_**18**_**]**^**3–**^)

In a 5 mL glass vial, (TBA)_4_[VW_5_O_19_] (50 mg, 0.022 mmol) was dissolved in MeCN (0.5 mL) forming a dark purple solution. A solution of 2,6-lutidinium tetrafluoroborate in MeCN (275 μL, 0.1 *M*, 0.028 mmol, 1.25 equiv) was added, upon which the mixture turned brown. The solution was stirred for 10 min and then placed inside a 20 mL scintillation vial. Diethyl ether was added to the outer 20 mL scintillation vial (until the solvent level of the diethyl ether was higher than the solvent level of the inner vial). The vial was sealed, and diethyl ether was allowed to diffuse into the inner vial over the course of 24 h. This led to the formation of large purple/brown crystals. The solvent was decanted, and the crystals were washed with 1:4 MeCN: Et_2_O (3 × 2 mL) and Et_2_O (3 × 2 mL). The crystals were then dried under vacuum. ^1^H NMR (500 MHz, CD_3_CN) δ 0.97 (s, 36 H), 1.38 (s, 24 H), 1.63 (s, 24 H), 3.15 (s, 24 H). ^51^V NMR (500 MHz, CD_3_CN) No signal. λ_max_ (MeCN) = 460 nm (ε_max_ = 258 M^–1^ cm^–1^). FTIR, ν_max_/cm^–1^: 3664(w), 2959, 2934, 2873, 1481(s), 1381, 1152(w), 1106(w), 1060(w), 1026(w), 990, 948(s), 884, 802(s), 766(s), 660, 591. Formula: (TBA)_3_[VW_5_O_19_H], Anal. Calcd for C_48_H_109_N_3_VW_5_O_19_ (mol. wt. 2002.544 g/mol): C, 28.79%; H, 5.49%; N, 2.10%. Found: C, 28.89%; H, 5.64%; N, 1.92%.

## Results and Discussion

### Redox Chemistry of **(TBA)**_**2**_**[W**_**6**_**O**_**19**_**]** and **(TBA)**_**3**_**[VW**_**5**_**O**_**19**_**]**

**Figure 2:**
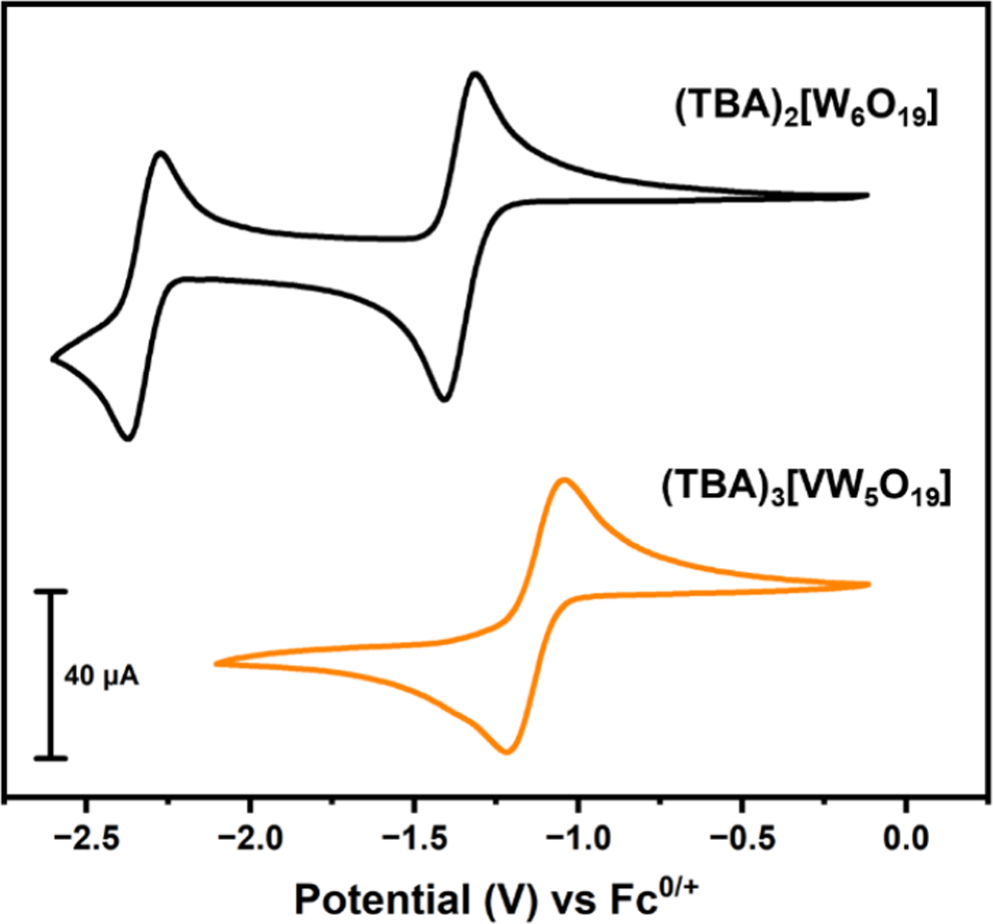
Cyclic voltammograms of 1 m*M* solutions of **[W**_**6**_**O**_**19**_**]**^**2–**^ (top, black) and **[VW**_**5**_**O**_**19**_**]**^**3–**^ (bottom, orange) collected in acetonitrile with 0.1 *M* TBA(PF_6_) as the supporting electrolyte (100 mV/s).

To frame our investigations of the proton-dependence of the redox properties of the vanadium-substituted polyoxotungstate, cyclic voltammetry (CV) experiments were performed in the *absence* of acid to gain an understanding of the electrochemistry of the POM in organic solvent. The CV of **[VW**_**5**_**O**_**19**_**]**^**3–**^ was collected in acetonitrile, with TBA(PF_6_) as the supporting electrolyte (100 mV/s; Figure 2), and compared to the previously reported CV of **[W**_**6**_**O**_**19**_**]**^**2–**^. The homometallic polyoxotungstate, **[W**_**6**_**O**_**19**_**]**^**2–**^, possesses two reversible reduction events (*E*_1/2_ = −1.36, – 2.32 V vs Fc^0/+^); data collected on the all-tungsten assembly matches those reported by Bond and co-workers.^45^ Addition of a third electron to **[W**_**6**_**O**_**19**_**]**^**2–**^ (i.e., **[W**_**6**_**O**_**19**_**]**^**4–**^ + 1e^–^ → **[W**_**6**_**O**_**19**_**]**^**5–**^) falls outside the electrochemical window of MeCN/TBA(PF_6_), however was observed during solid state CV of (TBA)_2_[W_6_O_19_] in the presence of an ionic liquid electrolyte (where the redox events are observed at more anodic potentials).^45^ Substitution of a single tungsten center by V^V^ yields drastic changes to the CV of the polyoxotungstate; in the voltammogram of **[VW**_**5**_**O**_**19**_**]**^**3–**^ only a single reduction event is observed within the electrochemical window of acetonitrile. The reduced number of redox processes is ascribed to the higher charge on the substituted polyoxotungstate, where addition of an electron to **[VW**_**5**_**O**_**19**_**]**^**3–**^ results in formation of **[VW**_**5**_**O**_**19**_**]**^**4–**^. The high charge on the relatively small heterometallic assembly renders addition of subsequent equivalents of electron density unfavorable due to electrostatic repulsion and therefore further redox events fall outside the electrochemical window of MeCN/TBA(PF_6_). This point was confirmed by the calculated redox potentials (see Table S8); the calculated second reduction of **[VW**_**5**_**O**_**19**_**]**^**3–**^ appears at −2.96 V vs Fc^0/+^.

The potential required for the one-electron reduction of **[VW**_**5**_**O**_**19**_**]**^**3–**^ is more positive than that of **[W**_**6**_**O**_**19**_**]**^**2–**^. We hypothesized that this is due to the fact that the vanadium dopant, a first-row transition metal, is more easily reduced than tungsten, as its empty d-orbitals are lower in energy. Similar observations have been noted previously in vanadium-substituted polyoxotungstates; in these types of heterometallic assemblies, distinct redox events shifted anodically from that of the tungsten-based reduction processes are observed.^46−49^ This reduction has been shown to be localized to the heterometal center in these examples (i.e., V^V^ + 1 e^–^ → V^IV^).

**Figure 3:**
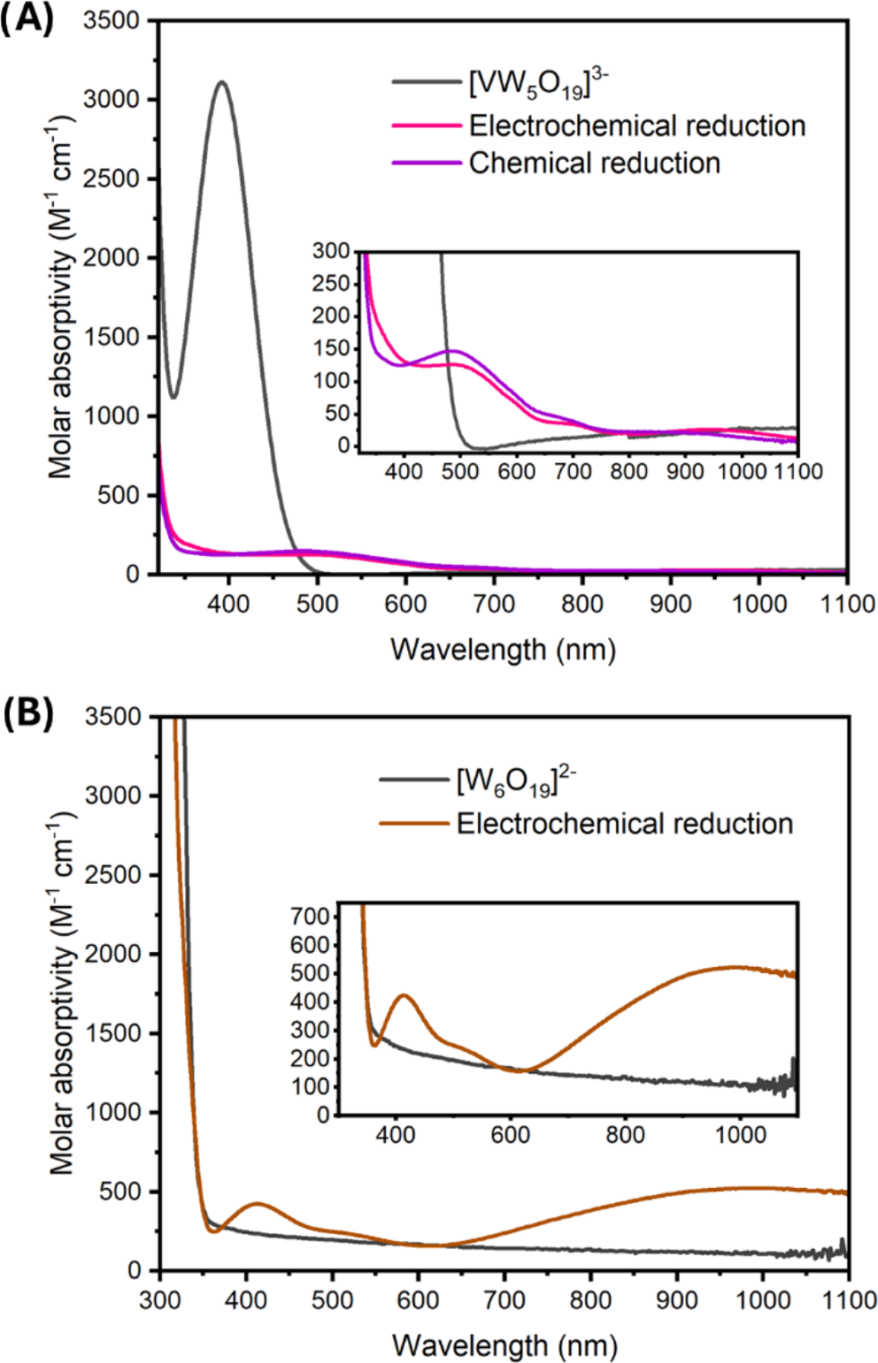
Electronic absorption spectra of (A) **[VW**_**5**_**O**_**19**_**]**^**3–**^ before (gray) and after reduction either electrochemically (pink) or chemically (purple) and (B) **[W**_**6**_**O**_**19**_**]**^**2–**^ before (gray) and after electrochemical reduction (brown). All spectra were collected in MeCN at room temperature (∼22 °C).

To confirm localization of electron density at vanadium upon reduction of **[VW**_**5**_**O**_**19**_**]**^**3–**^, our team studied the one-electron reduction of the heterometallic assembly compared to the isopolytungstate. First, bulk electrolysis of a 1 mM solution of **[VW**_**5**_**O**_**19**_**]**^**3–**^ in MeCN (0.1 M TBA(PF_6_) supporting electrolyte) at −1.39 V was performed (Figure S20). This led to a gradual color change from yellow to red/purple as the POM was reduced. The formation of **[VW**_**5**_**O**_**19**_**]**^**4–**^ was initially probed by CV and electronic absorption spectroscopy. The CV obtained after bulk electrolysis (Figure S21) was close to identical to that of **[VW**_**5**_**O**_**19**_**]**^**3–**^, with the only difference being the change in the open circuit potential (OCP), which had shifted to −1.20 V post electrolysis (−0.78 V prior to electrolysis). The electronic absorption spectrum obtained after bulk electrolysis is shown in Figure 3A. Fully oxidized **[VW**_**5**_**O**_**19**_**]**^**3–**^ possesses a sharp absorption feature at 392 nm (ε_max_ ≈ 3100 M^–1^ cm^–1^) which can be assigned to a O → V^V^ charge transfer. Upon reduction of the assembly, this feature no longer observed, which can be attributed to the fact that the energy of O → V^IV^ charge transfer is significantly blue-shifted. Analogous behavior has previously been observed for vanadium(IV)/(V) acetylacetonate compounds.^50^ The three broad absorptions observed at approximately 500 (ε_max_ = 153 M^–1^ cm^–1^), 700 nm (ε_max_ = 40 M^–1^ cm^–1^) and 910 nm (ε_max_ = 17 M^–1^ cm^–1^) can be compared with similar absorptions previously assigned to V^IV^ → W^VI^ IVCT and V^IV^ d–d transitions, respectively, in other vanadium containing polyoxotungstates, which supports the formal reduction of the V^V^ center to V^IV^, as opposed to a tungsten based reduction.^49,51^

Performing the same experiment on **[W**_**6**_**O**_**19**_**]**^**2–**^ gave different results. Bulk electrolysis of a 1 mM solution of **[W**_**6**_**O**_**19**_**]**^**2–**^ in MeCN (0.1 M TBA(PF_6_) supporting electrolyte) at −1.64 V (Figure S18) led to formation of a brown solution as the cluster was reduced (the fully oxidized material is colorless). The CV obtained post bulk electrolysis (Figure S19) was unchanged apart from the shift in the OCP from −0.56 to −1.39 V, consistent with one-electron reduction of the framework. The electronic absorption spectra of **[W**_**6**_**O**_**19**_**]**^**2–**^ obtained both before and after reduction are shown in Figure 3B. Both spectra possess intense absorption below 350 nm, which has previously been assigned to charge transfer (O → W ligand to metal charge transfer, LMCT) in both [W_10_O_32_]^4–^ and [XW_12_O_40_]^n–^ clusters.^52^ After electrochemical reduction, additional broad absorptions are observed at 412 nm (ε_max_ = 420 M^–1^ cm^–1^), 510 nm (ε_max_ = 240 M^–1^ cm^–1^), and 984 nm (ε_max_ = 520 M^–1^ cm^–1^). The lower wavelength absorptions are tentatively assigned to d–d transitions of the W^V^ center produced upon reduction, with the exact position and intensity of these bands likely related to the extent of delocalization of the added electron density.^52^ The more intense broad absorption centered at 985 nm can be confidently assigned to W^V^ → W^VI^ intervalence charge transfer (IVCT), with similar features observed upon reduction of other polyoxotungstates.^53,54^ Together these results support the formation of a delocalized ground state upon reduction of **[W**_**6**_**O**_**19**_**]**^**2–**^, with the “W^V^” character shared between the metal centers of the Lindqvist framework. Incorporation of a single vanadium center into the framework appears to shift the LUMO in such a way that reduction occurs preferentially at vanadium.

**Scheme 1:**
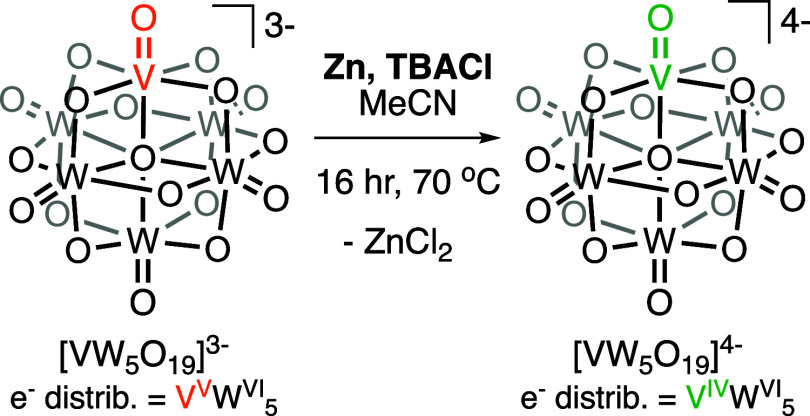
Synthesis of [VW_5_O_19_]^4–^

Chemical reduction of **[VW**_**5**_**O**_**19**_**]**^**3–**^on preparatory scale was also pursued. Previously our team has reported facile one-electron reduction of (TBA)_3_[PW_12_O_40_] by addition of one equivalent of TBA(BH_4_), showing the utility of this reagent for the reduction of polyoxotungstates. However, when one equivalent of TBA(BH_4_) was added to **[VW**_**5**_**O**_**19**_**]**^**3–**^, only a slight color change of yellow to brown was noted after extended reaction times (2 days), failing to reproduce the red/purple color observed during electrochemical reduction. Characterization of the products of these reactions only ever gave evidence of unreacted **[VW**_**5**_**O**_**19**_**]**^**3–**^. Instead, **[VW**_**5**_**O**_**19**_**]**^**3–**^ was treated with one equivalent of TBA(Cl) and an excess of zinc metal. The mixture was then heated at 70 °C for 16 h, resulting in the formation of a red/purple solution consistent with the reduction of **[VW**_**5**_**O**_**19**_**]**^**3–**^ to **[VW**_**5**_**O**_**19**_**]**^**4–**^ (Scheme 1). Following workup (see Experimental Section for details), single crystal X-ray diffraction quality crystals were obtained by vapor diffusion of Et_2_O into a saturated solution of the crude product dissolved in MeCN.

Diffraction experiments confirmed the presence of four TBA cations per [VW_5_O_19_] anion in the crystalline material (Figure S31, Table S4). The cluster crystallizes with two TBA cations in general positions and half of the tetraanionic **[VW**_**5**_**O**_**19**_**]**^**4–**^ cluster centered on a crystallographic inversion center that coincides with the central μ_6_-oxo. Unfortunately, the vanadium center is disordered across the three unique metal positions (total occupancy was constrained to 2.5 W and 0.5 V over the three positions, giving the 5:1 W/V ratio upon applying the crystallographic inversion center) which limits an in depth analysis of bond lengths, as the M–O bonds length are likely based on the weighted averages of the occupancies of the metal sites. The formulation of (TBA)_4_[VW_5_O_19_] was further confirmed by elemental analysis.

Further characterization of **[VW**_**5**_**O**_**19**_**]**^**4–**^ by CV and electronic absorption spectroscopy gave results that were consistent to those obtained from bulk electrolysis experiments. The CV of **[VW**_**5**_**O**_**19**_**]**^**4–**^ was very similar to pristine **[VW**_**5**_**O**_**19**_**]**^**3–**^, with the only major difference being the OCP, which was observed at −1.20 V. The obtained UV–vis absorption spectrum is shown in Figure 3A, and matches the spectrum obtained for the reduced heterometallic assembly accessed via bulk electrolysis.

**Figure 4:**
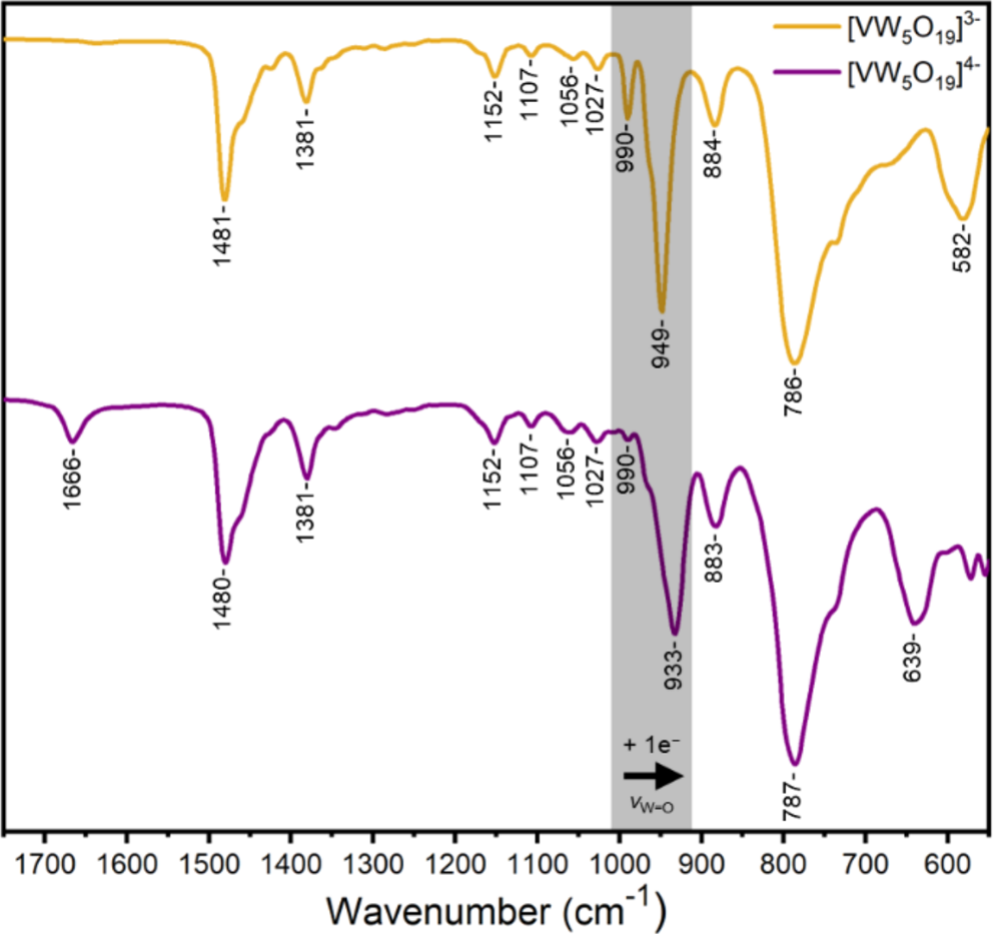
Infrared spectra of crystalline **[VW**_**5**_**O**_**19**_**]**^**3–**^ (yellow) and **[VW**_**5**_**O**_**19**_**]**^**4–**^ (purple).

The ability to isolate **[VW**_**5**_**O**_**19**_**]**^**4–**^ chemically also allowed characterization by infrared and NMR spectroscopy. The infrared spectrum, shown in Figure 4, is subtly different to that of the oxidized material. The major difference is a shifting of an intense peak at 949 cm^–1^ in **[VW**_**5**_**O**_**19**_**]**^**3–**^ to 933 cm^–1^ in **[VW**_**5**_**O**_**19**_**]**^**4–**^. This peak is assigned to the terminal ν (W=O) stretching mode and is reported to be responsive to changes in the charge on the Lindqvist unit, with the wavenumber decreasing as the negative charge increases.^55^ DFT also predicts a decrease in wavenumber of the terminal ν (W=O) stretching vibration from 997 cm^–1^ for **[VW**_**5**_**O**_**19**_**]**^**3–**^ to 976 cm^–1^ in **[VW**_**5**_**O**_**19**_**]**^**4–**^ (see Table S7). Therefore, the observed shift in the ν (W=O) stretching frequency supports the addition of an electron to the framework. The less intense peak at 990 cm^–1^ in the spectrum of **[VW**_**5**_**O**_**19**_**]**^**3–**^ can be assigned to a ν (V=O) stretching mode, previously observed at 989 cm^–1^ by Dmitrenko and co-workers.^56^ Calculated infrared (IR) spectra for **[VW**_**5**_**O**_**19**_**]**^**3–**^ indicate the terminal ν (V=O) stretching vibration at 1066 cm^–1^, while after reduction, this vibration shifts to a lower wavenumber of 1031 cm^–1^ for **[VW**_**5**_**O**_**19**_**]**^**4–**^. This peak is extremely weak after reduction. Therefore, the observed ν (V=O) stretching mode for **[VW**_**5**_**O**_**19**_**]**^**4–**^ is likely to appear under the broad ν (W=O) peak at 933 cm^–1^. ^1^H NMR spectroscopy revealed, as expected, only broad peaks assigned to the TBA cations (Figure S4). No signal was observed in the ^51^V NMR spectrum of **[VW**_**5**_**O**_**19**_**]**^**4–**^ (Figure S5); this is consistent with the formation of a localized, paramagnetic, V(IV) (d^1^) center.^49^

**Figure 5:**
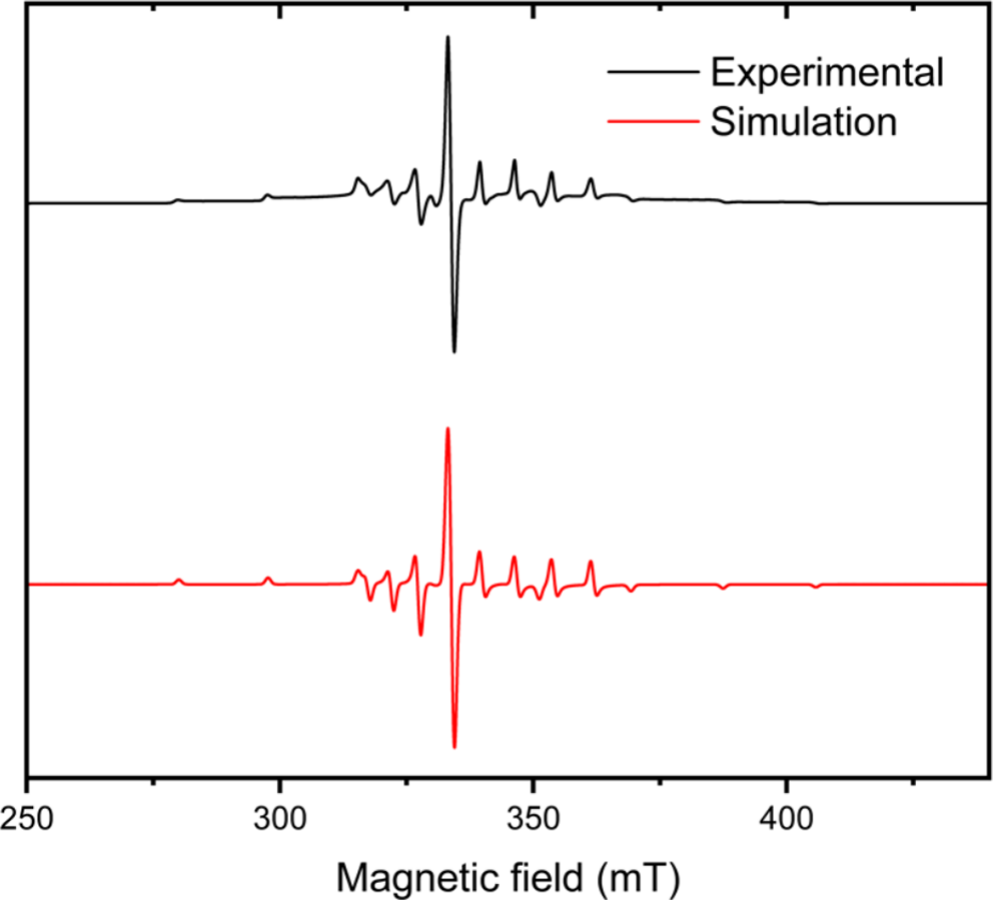
Ten K X-band EPR spectrum of **[VW**_**5**_**O**_**19**_**]**^**4–**^ in MeCN solution containing 0.1 M TBA(PF_6_) (black line) and simulated spectrum (red line). Simulation parameters are g_1_ = 1.968, g_2_ = 1.953, A(V) = [172.71, 491.10] MHz and lw (Gaussian) = 1.06 mT.

To unambiguously confirm the localization of the reduction at the vanadium center in the V-doped polyoxotungstate cluster, electron paramagnetic resonance (EPR) spectroscopy was conducted on the **[VW**_**5**_**O**_**19**_**]**^**4–**^ complex. The 10 K EPR spectrum of a frozen solution was recorded following the one-electron electrochemical reduction of **[VW**_**5**_**O**_**19**_**]**^**3–**^ (Figure 5). The axial spectrum is characterized by g_1_ = 1.968 and g_2_ = 1.953 and also exhibits hyperfine splitting attributed to the abundant ^51^V isotope (nuclear spin I = 7/2, natural abundance 99.75%), with hyperfine coupling constants of A(V) = [172.71, 491.10] MHz. The observed spectrum is similar to those previously reported spectra for one-electron reduced vanadium-doped Keggin-type polyoxometalates of the general formula [EVM_11_O_40_]^X–^ (where E = S, P, As, or V and M = Mo or W), further confirming reduction at vanadium.^12,46,57^ For comparison, a frozen solution EPR spectrum was recorded for the electrochemically reduced **[W**_**6**_**O**_**19**_**]**^**3–**^ complex. While the spectrum showed a mixture of two species, the major component has resonances at g_1_ = 1.759, g_2_ = 1.702 and A(W) = [0.371 0.177] MHz (Figure S34) consistent with the formation of **[W**_**6**_**O**_**19**_**]**^**3–**^.^58^ This indicates the localization of the unpaired electron at the tungsten center in contrast to the vanadium centered reduction observed for **[VW**_**5**_**O**_**19**_**]**^**4–**^.

### Density Functional Theory Calculations to Rationalize the Redox Properties of **[MW**_**5**_**O**_**19**_**]**^***n*–**^ Clusters (M = W, *n* = 2; M = V, *n* = 3)

The experiments described above provide insight into how incorporation of a heterometal in Lindqvist-type polyoxotungstates leads to changes in the redox properties. Density functional theory calculations can be used to provide additional perspective into the electronic structure of the homo- and heterometallic polyoxotungstates to further rationalize the observed discrepancies in analytical data.

**Figure 6:**
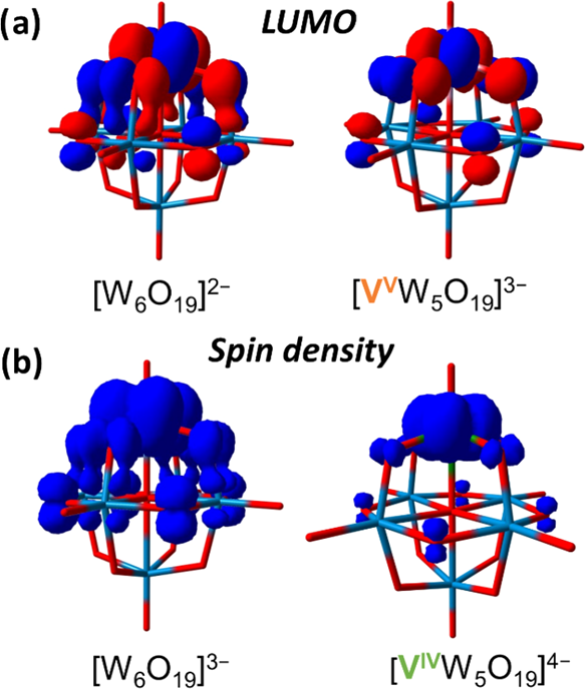
**(a)** Lowest unoccupied molecular orbitals of [MW_5_O_19_]^n–^ anions (M = W, *n* = 2; M = V, *n* = 3). **(b)** Spin density representations for the one-electron reduced analogues of [MW_5_O_19_]^n–^ anions (M = W, *n* = 3; M = V, *n* = 4).

Calculations show that the nature of the lowest unoccupied molecular orbital (LUMO) is critical for our understanding of the 1-electron reduction chemistry of the homo- and heterometallic polyoxotungstates (Figure 6). The visual representation of LUMO of each cluster in the series [MW_5_O_19_]^n–^ (M = W, *n* = 2; M = V, *n* = 3) is shown in Figure 6a. There is significant variation in the nature of the LUMO when a heterometal is present within the assembly. As expected, the LUMO of **[W**_**6**_**O**_**19**_**]**^**2–**^ is delocalized across multiple metal centers of the Lindqvist ion, primarily possessing tungsten d_*xy*_ character. The observed delocalization of electron density is consistent with the absorption spectrum of the reduced assembly; IVCT bands located at 984 nm suggest significant electronic communication between W^V^ (d^1^) and W^VI^ (d^0^) centers. Upon incorporation of a vanadium center into the Lindqvist framework, localization of a substantial portion of the LUMO is observed on the heterometal. This is a result of the presence of low-energy valence 3d orbitals for the vanadium center.

The primary location of reduction is further supported through calculation of the spin density plots of reduced analogues of the homo- and heterometallic assemblies (Figure 6b). The optimized geometries of the one-electron reduced anions, [MW_5_O_19_]^n–^ (M = W, *n* = 3; M = V, *n* = 4) were first calculated and spin density maps were then generated, revealing differences between α and β electron densities. In the case of the all-tungsten assembly, the spin density map of the reduced cluster, **[W**_**6**_**O**_**19**_**]**^**3–**^, reveals substantial delocalization of electron density across the framework. This observation is consistent with the calculated LUMO of the fully oxidized assembly and the experimental data described above. In the case of the vanadium-substituted anion, the obtained spin density map provides a clear visual indication in the electronic differences between the vanadium-doped and the undoped polyoxotungstates. In the case of **[VW**_**5**_**O**_**19**_**]**^**4–**^, there is substantial localization of the added electron density on the heterometal center. This is consistent with the obtained CV data (Figure 2), where **[VW**_**5**_**O**_**19**_**]**^**3–**^ was found to be more readily reducible than **[W**_**6**_**O**_**19**_**]**^**2–**^, which is only possible due to the availability of vanadium’s low lying empty 3d orbitals (as visualized in the LUMO and SOMO of the vanadium-doped cluster).

### Implications of a Vanadium Dopant on H Atom Uptake

**Figure 7:**
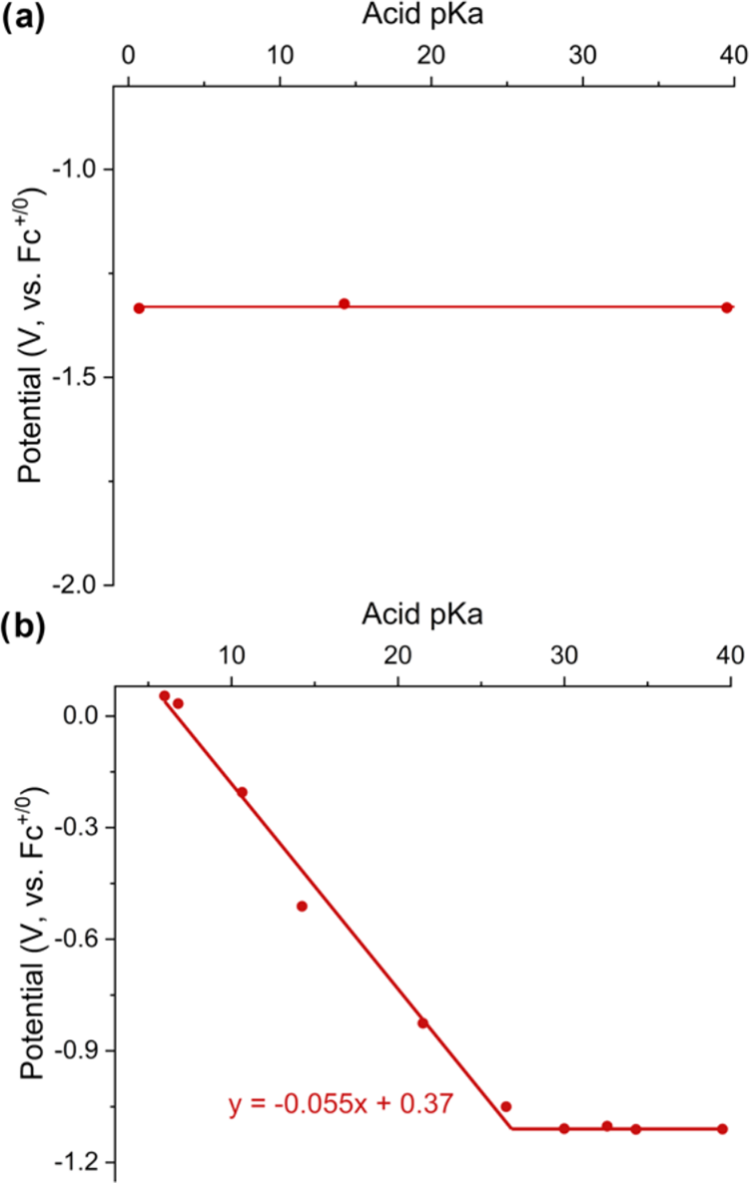
Potential-p*K*_a_ diagrams for (a) **[W**_**6**_**O**_**19**_**]**^**2–**^ (top) and (b) **[VW**_**5**_**O**_**19**_**]**^**3–**^ (bottom). Data points represent the measured reduction potential of 1 m*M* [POM]^n–^ in acetonitrile in the presence of 2 m*M* of various organic acids and supporting electrolyte (0.1 *M* TBA(PF_6_)). Reduction potentials are plotted versus the p*K*_a_ of the organic acid used in each experiment. The horizonal lines represent the acid-independent regions of the diagram, while the diagonal line represents the region of the diagram where the redox events are dependent on the strength of the acid added.

With an understanding of the electronic structure and redox properties of the vanadium-substituted polyoxotungstate in hand, we turned our attention to the elucidation of the thermochemistry of H atom uptake in these systems. Toward this goal, we analyzed the electrochemical properties of **[W**_**6**_**O**_**19**_**]**^**2–**^ and **[VW**_**5**_**O**_**19**_**]**^**3–**^ in acetonitrile in the presence of organic acids (2 equiv). We chose organic acids with p*K*_a_ values ranging from 0 to 40 (for full list of organic acids, see Figure S24/Table S2 in the Supporting Information file). Following the approach outlined by Dempsey and co-workers, we plotted the *E*_1/2_ value of the first reduction event of the POM as a function of the acidity of the organic acid added to construct a potential-p*K*_a_ diagram for **[W**_**6**_**O**_**19**_**]**^**2–**^ and **[VW**_**5**_**O**_**19**_**]**^**3–**^. These data are summarized in Figure 7.

To assess the acid dependent redox chemistry of the homometallic polyoxotungstate, a potential-p*K*_a_ diagram was constructed of **[W**_**6**_**O**_**19**_**]**^**2–**^ (Figure 7a). The cyclic voltammograms collected in the presence of organic acids with p*K*_a_ values ranging from 0 to 40 revealed no observed shift in the *E*_1/2_ of the first reduction process, suggesting the one-electron reduction of the cluster is not coupled to the addition of a proton under the prescribed reaction conditions.

In contrast, the electrochemistry of **[VW**_**5**_**O**_**19**_**]**^**3–**^ is quite sensitive to protons. As described above, the CV of **[VW**_**5**_**O**_**19**_**]**^**3–**^ exhibits a single reversible redox event in the absence of protons (V^V^ + 1 e^–^ → V^IV^; *E*_1/2_ = 1.13 V vs Fc^+/0^, Figure 2). Upon the addition of organic acids with p*K*_a_ values >26.5, the reduction event of **[VW**_**5**_**O**_**19**_**]**^**3–**^ is almost identical to that observed in the absence of protons. This observation suggests that both the oxidized and reduced charge states of the vanadium-doped polyoxotungstate are not sufficiently basic to deprotonate these weak acids. In contrast, the addition of organic acids with p*K*_a_ values <26.5 results in a gradual broadening and anodic shift in the reduction event of **[VW**_**5**_**O**_**19**_**]**^**3–**^, consistent with interactions between the anion and protons in solution. More potent organic acids result in more substantial shifts in the *E*_1/2_ value (*E*_1/2_ values summarized in Table S3). A plot of the p*K*_a_ of organic acid vs the *E*_1/2_ value of reveals a linear correlation between these two parameters; the slope of the line (55 mV/p*K*_a_ unit) is near the theoretical value of 59 mV/p*K*_a_ unit, consistent with a 1-electron, 1-proton transfer process at the surface of the anion. Using the intersections of acid dependent and independent events, the p*K*_a_ value for the reduced and protonated form of the polyoxotungstate, **[VW**_**5**_**O**_**18**_**(OH)]**^**3–**^, can be determined. This graphical approach yields a p*K*_a_ of 26.8.

**Scheme 2:**
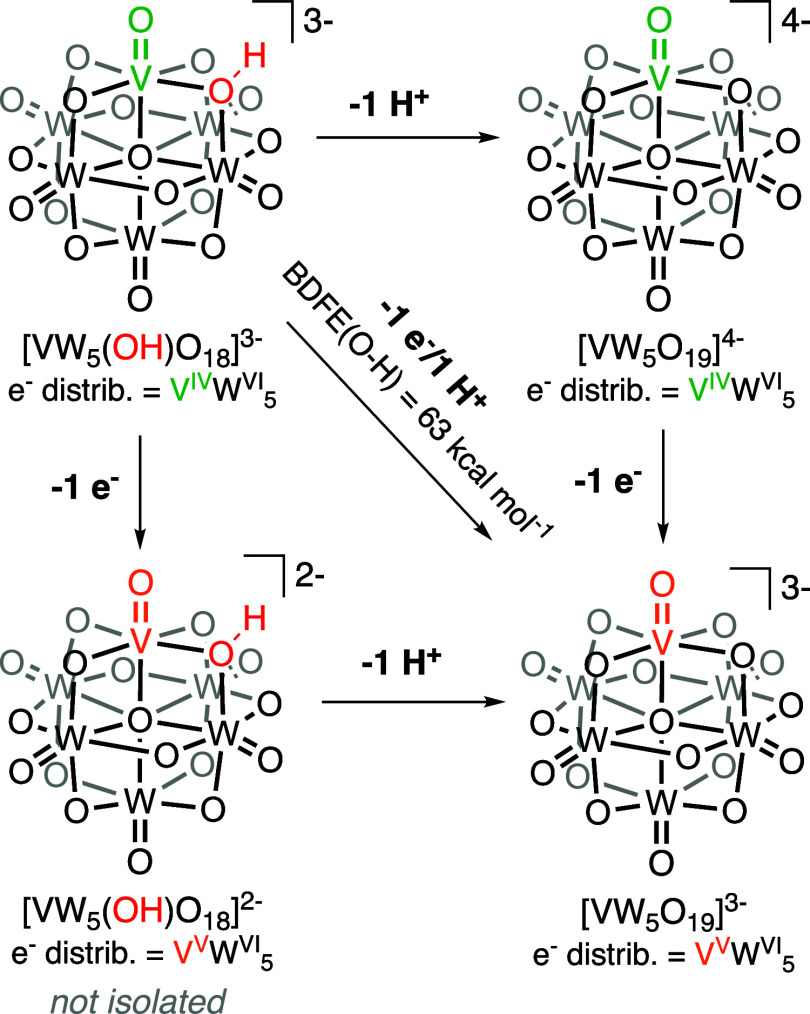
Square Scheme for the 1e^–^/1H^+^ Oxidation of **[VW**_**5**_**(OH)O**_**18**_**]**^**3–**^

With the reduction potential and p*K*_a_ value of the reduced cluster in hand, the thermochemistry of H atom uptake can be determined using the Bordwell eq (eq 1, Scheme 2).
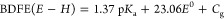
1Where p*K*_a_ and *E*^0^ are the experimentally determined parameters, and *C*_g_ is a constant associated with the reduction of H^+^ to H in acetonitrile (*C*_g_ = 52.6 kcal/mol). Complex **[VW**_**5**_**O**_**18**_**(OH)]**^**3–**^ has a BDFE(O–H) value of 63.8 kcal/mol in acetonitrile (Scheme 2).

To independently confirm the experimentally determined BDFE(O–H) of **[VW**_**5**_**O**_**18**_**(OH)]**^**3–**^, the reactivity of the oxidized POM with an H atom transfer reagent was explored. We selected 1,4-napthalenediol (H_2_NQ) as an optimal substrate, given that the O–H bonds of this reagent are comparable in strength to that measured for **[VW**_**5**_**O**_**18**_**(OH)]**^**3–**^ BDFE(O–H)_avg, MeCN_ = 63.3 kcal mol^–1^ for H_2_NQ (see Table S1 and following notes); BDFE(O–H)_MeCN_ = 63.8 kcal mol^–1^ for **VW**_**5**_**O**_**18**_**(OH)**^**3–**^). Addition of half an equivalent of H_2_NQ to **[VW**_**5**_**O**_**19**_**]**^**3–**^ results in the formation of a mixture of the oxidized and reduced versions of the POM and organic substrate, as determined by ^1^H NMR spectroscopy. The observed equilibrium indicates that the affinities of H atoms for both substrate and POM surface are identical (e.g., adjusted BDFE(O–H)_avg_ of H_2_NQ = BDFE(O–H) of **[VW**_**5**_**O**_**18**_**(OH)]**^**3–**^). By determining the concentration of reduced and oxidized substrate, we use a modified form of the Nernst equation to calculate the BDFE(O–H) of the surface hydroxide group of **[VW**_**5**_**O**_**18**_**(OH)]**^**3–**^ (eq 2, see Experimental Section for details)
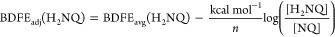
2where BDFE_adj_(H_2_NQ) is the average BDFE(N–H) of the reduced organic substrate adjusted for the relative concentrations of the reduced and oxidized organic compound in solution, BDFE(H_2_NQ) is the value reported in MeCN (63.3 kcal mol^–1^), *n* is the number of H atom equivalents transferred (*n* = 2 for NQ/H_2_NQ), and [H_2_NQ] and [NQ] are the concentrations of the reduced and oxidized versions of the substrate, respectively. Equilibrium experiments were repeated in triplicate (Figures S8–S10, Table S1). The reactions were left to stand for 21 days to ensure equilibrium was reached, however relative concentrations of reduced and oxidized substrates remained constant after 24 h. Through this form of analysis, an experimentally obtained BDFE(O–H) value of 63.0 ± 0.4 kcal mol^–1^ for complex **[VW**_**5**_**O**_**18**_**(OH)]**^**3–**^ was determined. This value closely resembles that obtained from the potential-p*K*_a_ diagram (*vide supra*), validating the thermochemistry of H atom uptake at the surface of the V-doped Lindqvist-type polyoxotungstate.

### Isolating One-Electron Reduced and Protonated **[VW**_**5**_**O**_**19**_**]**^**3–**^

The experimentally determined BDFE(O–H) value of **[VW**_**5**_**O**_**18**_**(OH)]**^**3–**^ (∼63 kcal mol^–1^) is similar to values for homometallic polyoxovanadates reported previously by members of our research team.^24^ We note that H atom uptake in these species proceeds via 2H^+^/2e^–^ processes; this is distinct from the reduction chemistry observed for **[VW**_**5**_**O**_**18**_**(OH)]**^**3–**^, which occurs via addition of a single proton–electron pair. In the case of H atom uptake at bridging oxide moieties located at the surface of a Lindqvist-type polyoxovanadate-alkoxide, BDFE(O–H)_avg_ values ranging from 61 to 66 kcal mol^–1^ were measured, with lower O–H bond strengths associated with reduced variants of the hexavanadate assembly (e.g., [V_6_^IV^(OH)_6_O_7_(TRIOL^R^)_2_]^2–^). Indeed, BDFE(O–H)_avg_ values of ∼66 kcal mol^–1^ were reported for high-valent forms of [V_2_^IV^V_4_^V^O_11_(OH)_2_(TRIOL^R^)_2_]^2–^ (R = CH_3_, NO_2_),^24^ which at surface level seem to most closely resemble the oxidation state distribution of **[VW**_**5**_**O**_**18**_**(OH)]**^**3–**^. We hypothesize that the lower BDFE(O–H) value is due to the increased acidity of the cluster surface; the μ_2_-O^2–^ moiety which hosts the H atom bridges a vanadium and tungsten center. The acidity of the tungstate moiety likely decreases the affinity of the proton for the surface of the reduced anion, modulating the strength of the O–H interaction.

During our group’s studies examining the PCET reactivity of (TBA)_3_[PW_12_O_40_] we were unable to isolate the reduced and protonated polyoxotungstate species. This was attributed to the very low BDFE(O–H)_avg_, which makes “(TBA)_3_[PW^V^(OH)W_11_^VI^O_39_]” unstable with respect to H_2_ evolution.^18^ Contrary to this, the vanadium-substituted Lindqvist structure discussed in this work possesses a relatively high BDFE(O–H) and therefore we would expect regeneration of oxidized **[VW**_**5**_**O**_**19**_**]**^**3–**^ via hydrogen evolution to be thermodynamically disfavored. This led us to pursue the isolation of the reduced and protonated species **[VW**_**5**_**O**_**18**_**(OH)]**^**3–**^. To do this, a purple solution **[VW**_**5**_**O**_**19**_**]**^**4–**^ (obtained via the chemical reduction of **[VW**_**5**_**O**_**19**_**]**^**3–**^ discussed above) was treated with 1.25 equiv of 2,6-lutidinium tetrafluoroborate (an acid with a p*K*_a_ < 26.8 was chosen based on results from potential-p*K*_a_ studies discussed above). This led to an immediate color change to brown. Vapor diffusion of diethyl ether directly into this mixture led to the formation of dark purple/brown crystals. These crystals were analyzed by SCXRD and were found to behave similarly to those of **[VW**_**5**_**O**_**19**_**]**^**4–**^ obtained in this work. The major difference was the presence of only three TBA cations per [VW_5_O_19_] anion, consistent with protonation of **[VW**_**5**_**O**_**19**_**]**^**4–**^ and successful formation of **[VW**_**5**_**O**_**18**_**(OH)]**^**3–**^. Unfortunately, the presence of an inversion center coincident with the central μ_6_-oxo leads to only three independent metal positions for the Lindqvist ion, with the vanadium center disordered over all positions. This not only prevents detailed bond length analysis, but also prevents localization of the site of protonation. To rule out the possibility of this crystalline material being that of oxidized **[VW**_**5**_**O**_**19**_**]**^**3–**^, which is also reported as a disordered structure with three TBA cations per Lindqvist POM, ^51^V NMR was recorded on the crystalline material. No peak was observed between 0 and −1500 ppm (**[VW**_**5**_**O**_**19**_**]**^**3–**^ has a single peak at −508 ppm shown in Figure S3) consistent with the presence of a paramagnetic V(IV) (d^1^) metal center, and therefore supporting the assignment of [**VW**_**5**_**O**_**18**_**(OH)]**^**3–**^.

**Figure 8:**
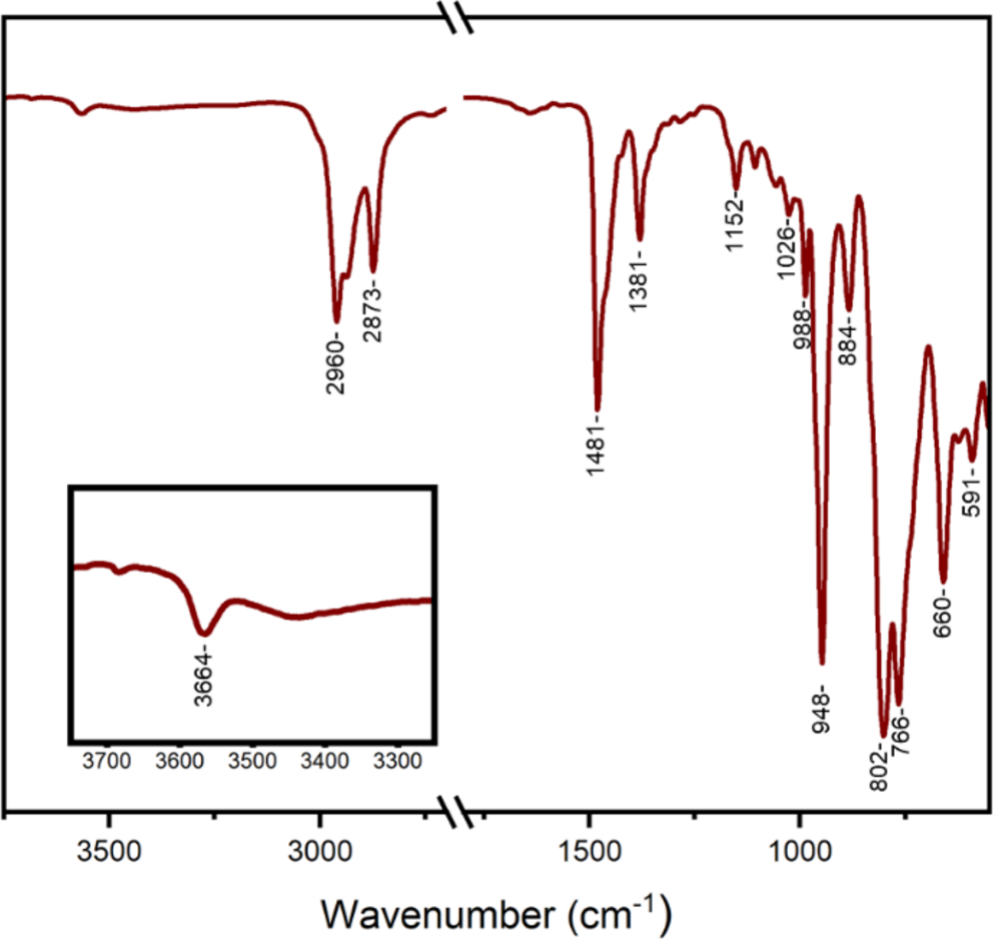
Infrared spectrum of **[VW**_**5**_**O**_**18**_**(OH)]**^**3–**^ obtained by treatment of [**VW**_**5**_**O**_**19**_**]**^**4–**^ with 2,6-lutidinium tetrafluoroborate.

To gain evidence of framework protonation, infrared spectroscopy was performed, and the resulting spectrum is shown in Figure 8. The spectrum is very similar to those shown in Figure 4 for **[VW**_**5**_**O**_**19**_**]**^**3–**^ and **[VW**_**5**_**O**_**19**_**]**^**4–**^. The terminal ν (W=O) stretching frequency is present at 948 cm^–1^, which is identical to that of oxidized **[VW**_**5**_**O**_**19**_**]**^**3–**^, consistent with a drop in charge from 4– to 3– upon protonation, supporting the assignment of **[VW**_**5**_**O**_**18**_**(OH)]**^**3–**^. This is further corroborated by the presence of a small peak at 3664 cm^–1^ which can be assigned to the O–H stretching frequency of V–O(H)-W. Prior to protonation, the infrared spectrum of **[VW**_**5**_**O**_**19**_**]**^**4–**^ features a single peak at 787 cm^–1^ (Figure 4) typical for bridging W–O–W stretching vibrations. This peak is split into two peaks for **[VW**_**5**_**O**_**18**_**(OH)]**^**3–**^, observed at 802 and 766 cm^–1^, respectively. Calculated infrared frequencies for **[VW**_**5**_**O**_**18**_**(OH)]**^**3–**^ confirm the appearance of this new peak, which is consistent with a drop in symmetry (from *C_4V_* to *C_s_*) upon protonation (see Table S7). The most intense calculated bridging W–O–W stretching for **[VW**_**5**_**O**_**19**_**]**^**4–**^ appears at 794 cm^–1^, which becomes split after protonation. It is worth mentioning that some of the additional peaks resulting from the drop in symmetry may not be visible in the experimental FT-IR due to the low IR intensity or because they are hidden under broad peaks. The sharp peak at 988 cm^–1^ can be assigned to the terminal ν (V=O) stretching mode.^56^ These findings, as well as elemental analysis data, all support the successful formation of **[VW**_**5**_**O**_**18**_**(OH)]**^**3–**^ upon treatment of **[VW**_**5**_**O**_**19**_**]**^**4–**^ with acid.

**Figure 9:**
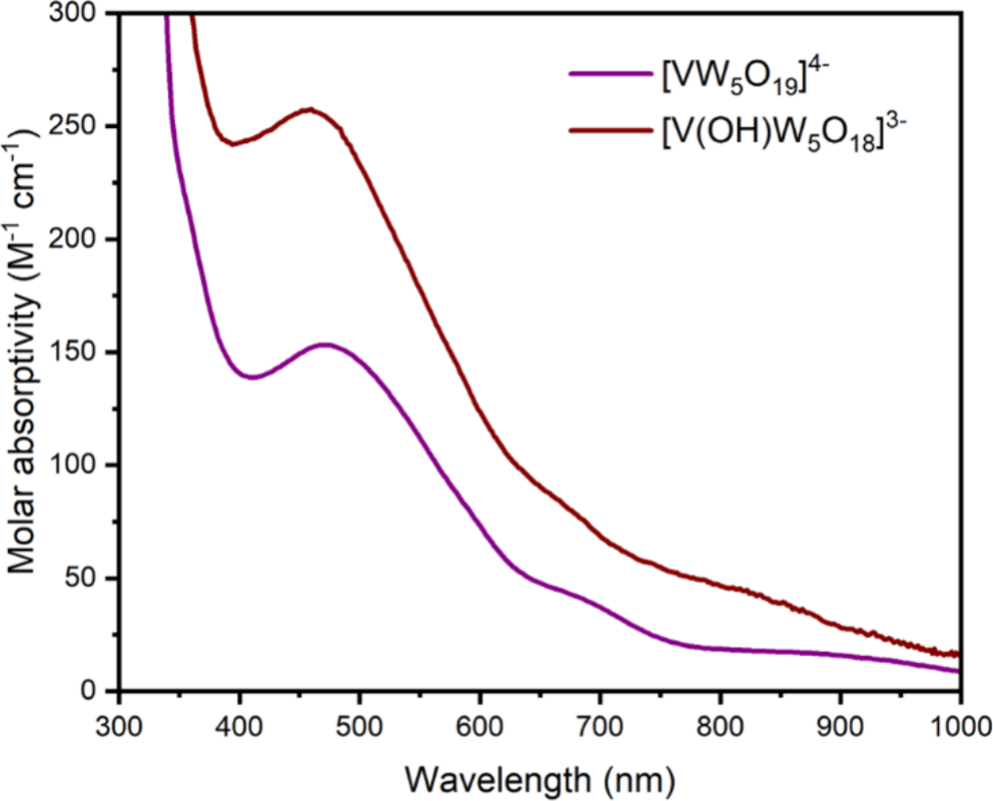
UV–vis spectrum of **[VW**_**5**_**O**_**18**_**(OH)]**^**3–**^ (brown) obtained by treatment of **[VW**_**5**_**O**_**19**_**]**^**4–**^ (purple) with 2,6-lutidinium tetrafluoroborate. Both spectra were obtained in MeCN.

Dissolution of crystalline **[VW**_**5**_**O**_**18**_**(OH)]**^**3–**^ in MeCN led to the formation of brown/purple solutions which appeared only subtly different from those of **[VW**_**5**_**O**_**19**_**]**^**4–**^, suggesting protonation has only a limited impact on the electronic structure of the reduced species. This behavior was investigated by electronic absorption spectroscopy (Figure 9). The spectrum obtained after reduction and protonation varies only slightly from that of the reduced material. The wavelength of the peak assigned to V^IV^ → W^VI^ IVCT changes from 472 nm in **[VW**_**5**_**O**_**19**_**]**^**4–**^ to 460 nm in **[VW**_**5**_**O**_**18**_**(OH)]**^**3–**^. There is also an apparent increase in molar absorptivity for the process with an ε_max_ = 153 M^–1^ cm^–1^ for **[VW**_**5**_**O**_**19**_**]**^**4–**^, compared to an ε_max_ = 258 M^–1^ cm^–1^ for **[VW**_**5**_**O**_**18**_**(OH)]**^**3–**^ obtained chemically. The shape of the rest of the spectrum is similar, though the broad absorption that trails across the visible region is always more intense for **[VW**_**5**_**O**_**18**_**(OH)]**^**3–**^ compared to **[VW**_**5**_**O**_**19**_**]**^**4–**^, which may help to account for the subtle color change toward brown.

Similar results were obtained electrochemically. Both bulk electrolysis at −1.39 V vs Fc^0/+^ followed by protonation with 2,6-lutidinium tetrafluoroborate or bulk electrolysis at −1.04 V vs Fc^0/+^ in the presence of protons yield solutions with open circuit potentials of −1.20 V and −0.71 V respectively (both more negative than the potential of the respective V^IV^/V^V^ redox couples, see Figures S25–S28) and electronic absorption spectra (Figures S29 and S30) in line with those shown in Figure 9.

**Figure 10:**
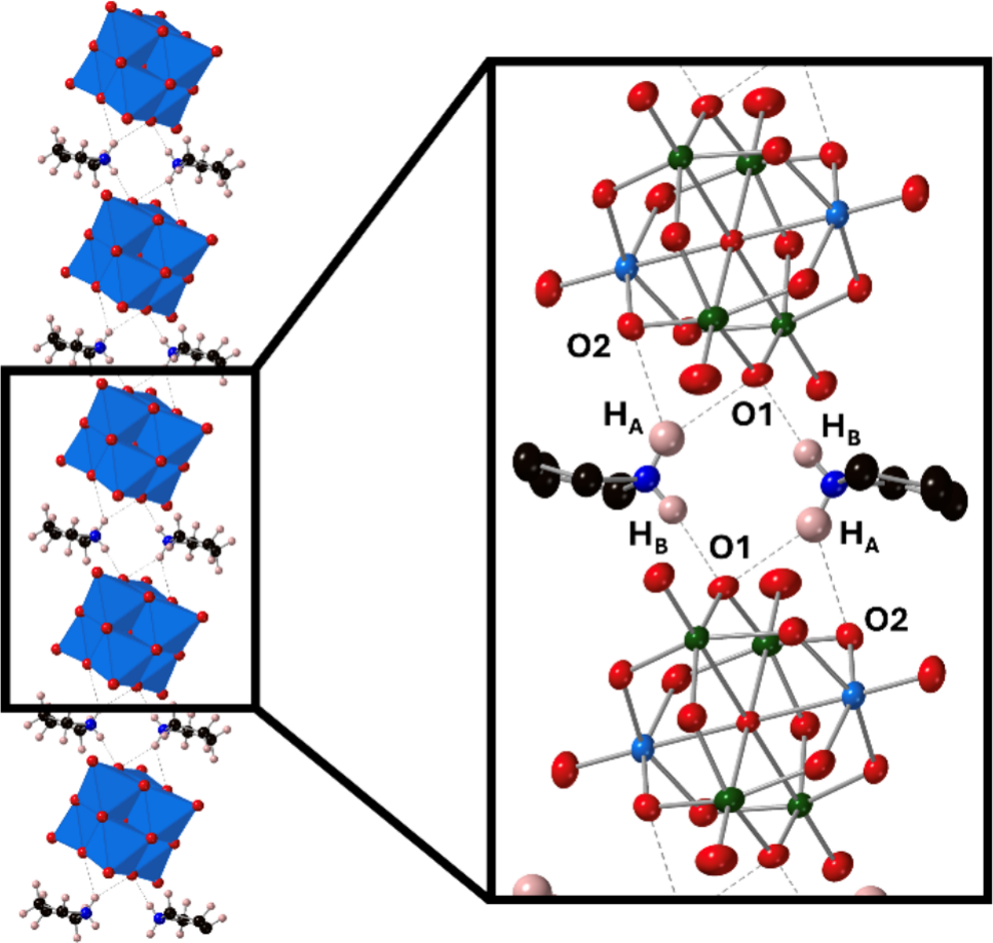
SCXRD structure of (TBA)_2_(PyrrH)_2_[VW_5_O_19_]. The extended chain-like structure is shown with [VW_5_O_19_]^4–^ (shown as blue polyhedra) units linked by pyrrolidinium cations. The cation–anion interactions are highlighted in the inset with blue metal sites only occupied by W and green sites having mixed W/V occupancy. TBA cations and some disorder of the pyrrole framework was omitted of clarity.

In attempts to obtain direct crystallographic evidence of protonation of the reduced **[VW**_**5**_**O**_**19**_**]**^**4–**^ framework, reactions with weaker acids (closer to the p*K*_a_ value of 26.8 obtained from out potential-p*K*_a_ experiments) were also carried out. Treatment of **[VW**_**5**_**O**_**19**_**]**^**4–**^ with triethylammonium tetrafluoroborate (Et_3_N p*K*_aH_ = 18.83 in MeCN), followed by diethyl ether vapor diffusion, gave only a structure identical to that previously obtained for **[VW**_**5**_**O**_**18**_**(OH)]**^**3–**^, indicating complete proton transfer and loss of TBA(BF_4_)/NEt_3_. However, upon treatment of **[VW**_**5**_**O**_**19**_**]**^**4–**^ with pyrrolidinium tetrafluoroborate (and vapor diffusion of diethyl ether) (pyrrolidine p*K*_aH_ = 19.62 in MeCN) a completely different structure was obtained and is shown in Figure 10. Instead of complete proton transfer, and concomitant loss of the conjugate base of the acid added, a mixed cation species which could be described as (TBA)_2_(PyrrH)_2_[VW_5_O_19_] (PyrrH = pyrrolidinium) was obtained. As for the other structures presented in this paper, the central μ_6_-oxo lies on a crystallographic inversion which leads to disorder in the occupancy of the metal sites. However, in this case the vanadium center is only disordered over two of the three unique metal sites present in asymmetric unit, with the total occupancy of vanadium coming to close to 0.5 per half cluster if not constrained (consistent with the 1 V to 5 W ratio expected). The relative localization of the vanadium center in this structure compared to the others discussed in this work, where vanadium is disordered over all unique metal sites, can likely be attributed to the hydrogen bonding interactions between the closely associated pyrrolidinium cations and the basic V–O–W bridging sites (See Figures S35 and S36).

Closer inspection of this interaction shows the formation of chains of anions linked by pyrrolidinium cations, with each –NH_2_ unit forming single shorter hydrogen bonding interaction with one anion (H_B_-O1 ca. 1.89 Å) and a longer pair of hydrogen bonding interactions with two oxygen atoms of another anion (H_A_-O1 and H_A_-O2 at ca. 2.17 and 2.37 Å respectively). The lack of any interaction between the pyrrolidinium and terminal M=O sites supports the hypothesis that these sites are less basic than the bridging sites in these systems, with the most basic sites likely being the oxygen atoms of the V–O–W bridges given the additional electron density added to the system upon reduction is localized at vanadium.

It is noteworthy that the SCXRD structure obtained here supports localization of the protons on pyrrolidine (found from the Fourier difference map and refined freely), rather than localization of the protons on the POM and subsequent formation of O–H···NHR_2_ type hydrogen bonds. When taken together with the results obtained from treatment of **[VW**_**5**_**O**_**19**_**]**^**4–**^ with triethylammonium tetrafluoroborate, it is apparent that changing the strength of the acid by less than a single p*K*_a_ unit leads to a switch from a structure containing **[VW**_**5**_**O**_**19**_**]**^**4–**^ (formally unprotonated) to those containing **[VW**_**5**_**O**_**18**_**(OH)]**^**3–**^. However, it is difficult to tell if the ability of pyrrolidinium to bridge **[VW**_**5**_**O**_**19**_**]**^**4–**^ units and form extended chains during lattice formation provides an additional driving force for localization of the proton on the organic group (meaning there are more factors to consider than simply the p*K*_a_ of the acid used).

### Density Functional Theory Calculations Investigating H Atom Uptake in **[VW**_**5**_**O**_**19**_**]**^**3–**^

**Figure 11:**
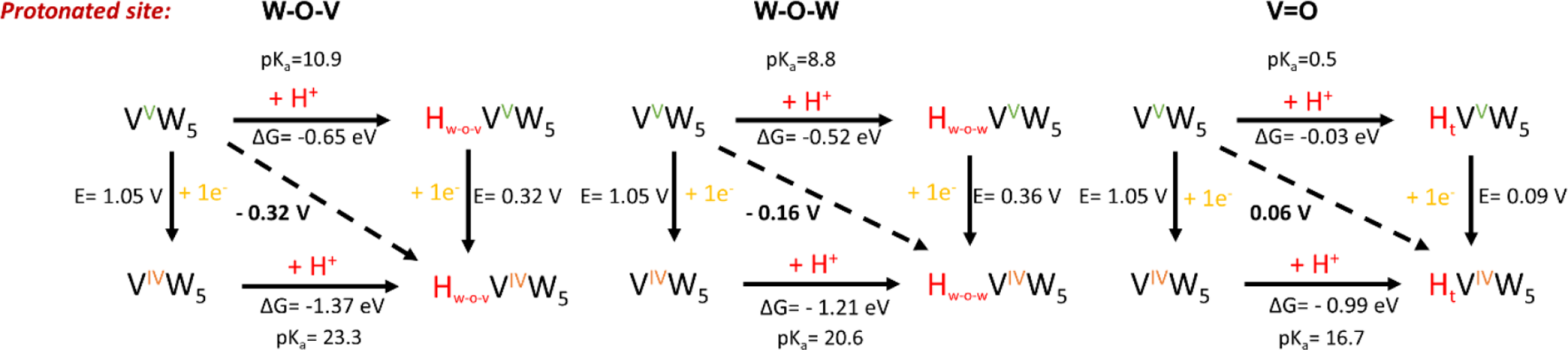
Calculated square schemes summarizing the PCET, PT and ET events for different protonation sites of **[VW**_**5**_**O**_**19**_**]**^**3–**^. Potentials are given in V vs Fc/Fc^+^ for electrochemical processes (PCET and ET), and the chemical processes related to proton transfers (PT) are expressed as free Gibbs energy (in eV) with corresponding p*K*_a_.

To better understand the behavior of **[VW**_**5**_**O**_**19**_**]**^**3–**^ upon reduction and the H atom uptake in the reduced **[VW**_**5**_**O**_**19**_**]**^**4–**^ framework, the reduction and protonation events likely to occur under electrochemical conditions for **[VW**_**5**_**O**_**19**_**]**^**3–**^ were investigated. Figure 11 shows the calculated square schemes, summarizing the possible electron transfers (ET), proton transfers (PT) and PCET events possible for **[VW**_**5**_**O**_**19**_**]**^**3–**^ depending on the protonation site (W–O–W, W–O–V and V=O) (see SI). The protonation of the terminal oxygen W=O was not considered due to its lower basicity compared to the other oxygen sites (Figures S35 and S36).

In this Figure, vertical processes correspond to reduction reactions (ET), horizontal ones to protonations (PT, i.e., simple acid–base reactions) and diagonal arrows represent PCET processes, corresponding to the addition of one electron and one proton. Figure 11 shows that the PCET event is a thermodynamically favorable step when the protonation occurs at either bridging W–O–V or W–O–W sites, leading to the reduced and protonated species **[VW**_**5**_**O**_**18**_**(OH)]**^**3–**^ (**HV**^**IV**^**W**_**5**_), however when protonation occurs at the terminal V=O site (targeting **H**_**t**_**V**^**IV**^**W**_**5**_) the PCET event is more energy demanding. The first ET to go from **[VW**_**5**_**O**_**19**_**]**^**3–**^ (**V**^**V**^**W**_**5**_) to **[VW**_**5**_**O**_**19**_**]**^**4–**^ (**V**^**IV**^**W**_**5**_) requires, according to calculations, 1.05 V which agrees with the experimental value (Figure 2). The PT to go from **V**^**IV**^**W**_**5**_ to **HV**^**IV**^**W**_**5**_ is thermodynamically favorable in all the cases due to the increase in the electron density of the system. The energy changes upon protonation of the W–O–W and W–O–V bridges of **V**^**IV**^**W**_**5**_ (i.e., giving **H**_**W–O–W**_**V**^**IV**^**W**_**5**_ or **H**_**W–O–V**_**V**^**IV**^**W**_**5**_) are −1.21 and −1.37 eV, respectively. The more negative energy change obtained upon protonation of the V–O–W bridging sites of **V**^**IV**^**W**_**5**_, and the associated calculated p*K*_a_, agrees well with the experimental p*K*_a_ obtained from electrochemical potential-p*K*_a_ experiments. Together with SCXRD results showing preferential formation of H-bonding interactions at the bridging positions of **[VW**_**5**_**O**_**19**_**]**^**4–**^, these results all point to the fact that the preferred protonation sites in this system are those directly adjacent to the vanadium dopant (i.e., W–O–V). Examination of the same protonation reactions for the oxidized **[VW**_**5**_**O**_**19**_**]**^**3–**^ gave consistent results, where protonation of the W–O–V (i.e., giving **H**_**W–O–V**_**V**^**V**^**W**_**5**_) bridging sites is found to be the most energetically favorable, though the cluster is much less basic overall. The subsequent ET from **HV**^**V**^**W**_**5**_ to **HV**^**IV**^**W**_**5**_ also become less energetically demanding due to the decrease of the total charge in the system.

## Conclusions

Herein, we describe the redox properties of vanadium-substituted Lindqvist-type polyoxotungstates. Electrochemical experiments performed on the series of POMs reveal that nature of the heterometal has a significant impact on the redox properties of the assembly. These observations are largely rationalized with the aid of density functional theory calculations, which indicate that the nature of the lowest unoccupied molecular orbital is influenced by the incorporation vanadium, such that reduction is localized at the heterometal center.

The electronic structure of the reduced vanadium-doped polyoxotungstate has a profound influence on the reactivity with H atom equivalents. In the case of the all-tungsten assembly, reduction is delocalized across tungstate moieties that compose the Lindqvist core; distribution of electron density and the inherent acidity of W^VI^ render these clusters inert to reactivity with H atom equivalents. In contrast, the localized redox chemistry of [VW_5_O_19_]^3–^ manifests in a sufficiently basic bridging oxide site positioned between the dopant and framework metals and construction of a potential-p*K*_a_ diagram reveals a BDFE(O–H) for the reduced and protonated assembly, **[VW**_**5**_**O**_**18**_**(OH)]**^**3–**^, of ∼63 kcal mol^–1^. The thermochemistry of the O–H bond of was confirmed through independent analysis. The relatively high basicity of the bridging oxo sites in the [VW_5_O_19_] framework imparts thermodynamically stability upon the reduced and protonated **[VW**_**5**_**O**_**18**_**(OH)]**^**3–**^ and thus we have reported the synthesis of this compound via stepwise reduction and protonation. DFT calculations and SCXRD experiments using acids with different p*K*_a_’s in the protonation step gave direct evidence that protonation occurs preferentially at the bridging oxo sites.

Heterometal substitution in polyoxometalates is a well-established technique for manipulating the electronic structure of the assembly.^12,13,59^ In this study, we demonstrate incorporation of a heterometal can be critical in determining the resultant reactivity of the assembly with H atom equivalents, “switching on” PCET reactivity in a Lindqvist-type polyoxotungstate. Affinity of the POM surface for H atom equivalents is only possible when a first-row transition metal is embedded within the assembly such that access to low-energy, 3d orbitals lowers the overpotential required for reduction and increases the basicity of the POM surface.

## Supporting Information Available

Additional characterization data of materials, crystallographic information, and details about computational methods used in this work (PDF)
