# Supplementary material for: Vanadium Substitution Dictates H Atom Uptake at Lindqvist-type Polyoxotungstates
Source: Inorg Chem. 2024 Nov 20;63(49):23304–16. doi: 10.1021/acs.inorgchem.4c03873 (PMC11632773; doi:10.1021/acs.inorgchem.4c03873)
Supplement: Supplementary file 1 — ic4c03873_si_001.pdf [file ic4c03873_si_001.pdf]

## Electronic Supporting Information

# Vanadium Substitution Dictates H-atom Uptake at Lindqvist-type Polyoxotungstates

Dominic Shiels[1,2], Zhou Lu[1], Magda Pascual-Borràs[2], Nathalia Cajiao[1,3], Thompson V. Marinho[1], William W. Brennessel[1], Micheal L. Neidig,[3] R. John Errington[2]*, and Ellen M. Matson[1]*

*[1] Department of Chemistry, University of Rochester, Rochester NY, 14627 USA*

*[2]NUPOM Lab Chemistry, School of Natural & Environmental Sciences, Newcastle University, Newcastle upon Tyne, NE1 7RU, UK*

*[3]Inorganic Chemistry Laboratory, Department of Chemistry, University of Oxford, South Parks Road, Oxford, OX1 3QR, UK*

**Corresponding Author Contact Information:**

Ellen M. Matson: matson@chem.rochester.edu

R. John Errington: john.errington@newcastle.ac.uk

**1. NMR SPECTRA** ........ **S3**
**2. INFRARED SPECTRA** ........ **S9**
**3. ELECTROCHEMISTRY** ........ **S11**
**5. ELECTRONIC ABSORPTION SPECTROSCOPY** ........ **S20**
**6. SINGLE CRYSTAL X-RAY DIFFRACTION DATA** ........ **S21**
**7. EPR SPECTRA** ........ **S27**
**8. COMPUTATIONAL METHODS AND CALCULATIONS** ........ **S28**
**9. REFERENCES** ........ **S35**

## 1. NMR spectra

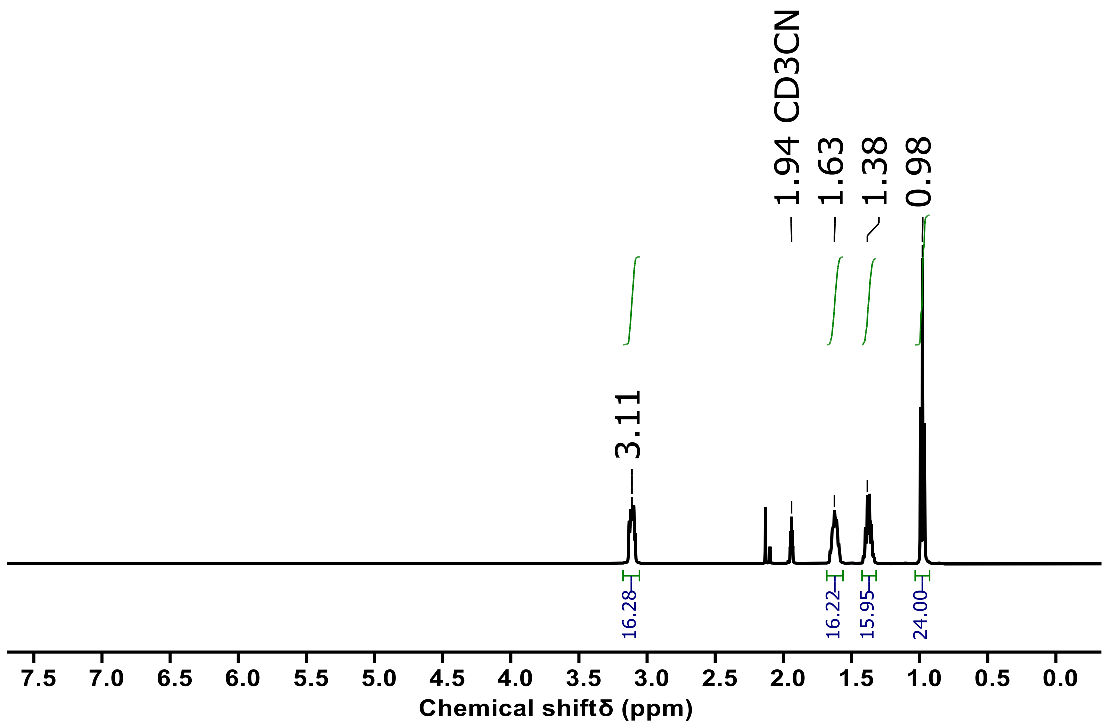


**Figure S1:** $^{1}H$ NMR spectrum (500 MHz) of $(TBA)_2[W_6O_{19}]$ in $CD_3CN$.

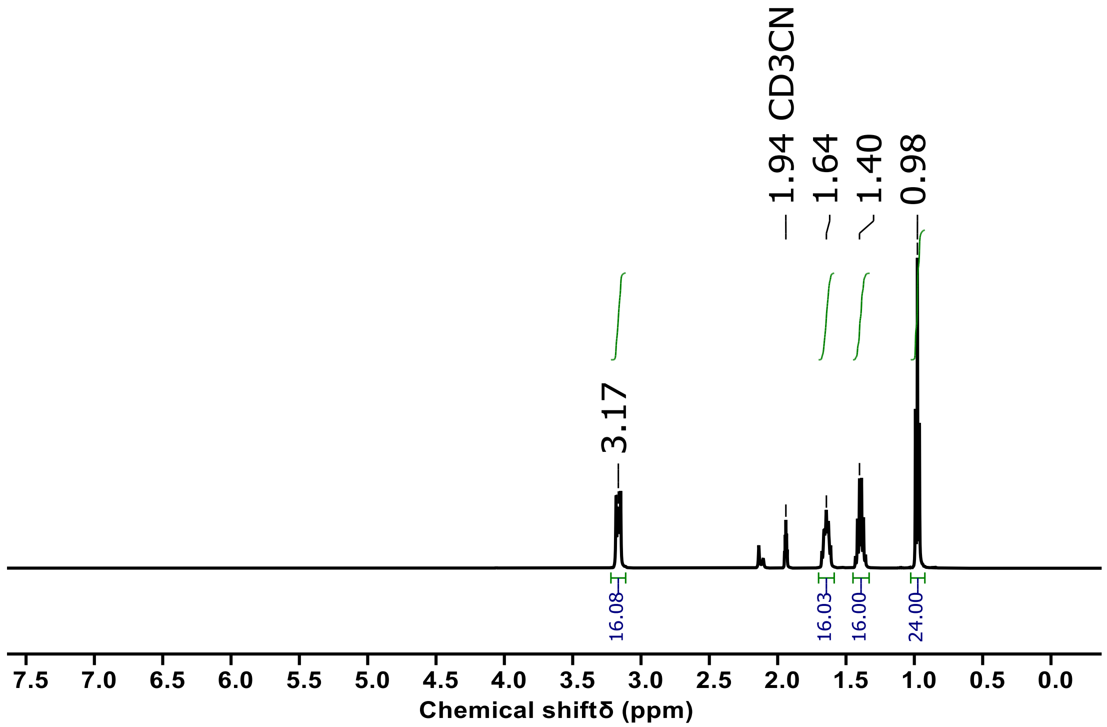


**Figure S2:** $^{1}H$ NMR spectrum (500 MHz) of $(TBA)_3[VW_5O_{19}]$ in $CD_3CN$.

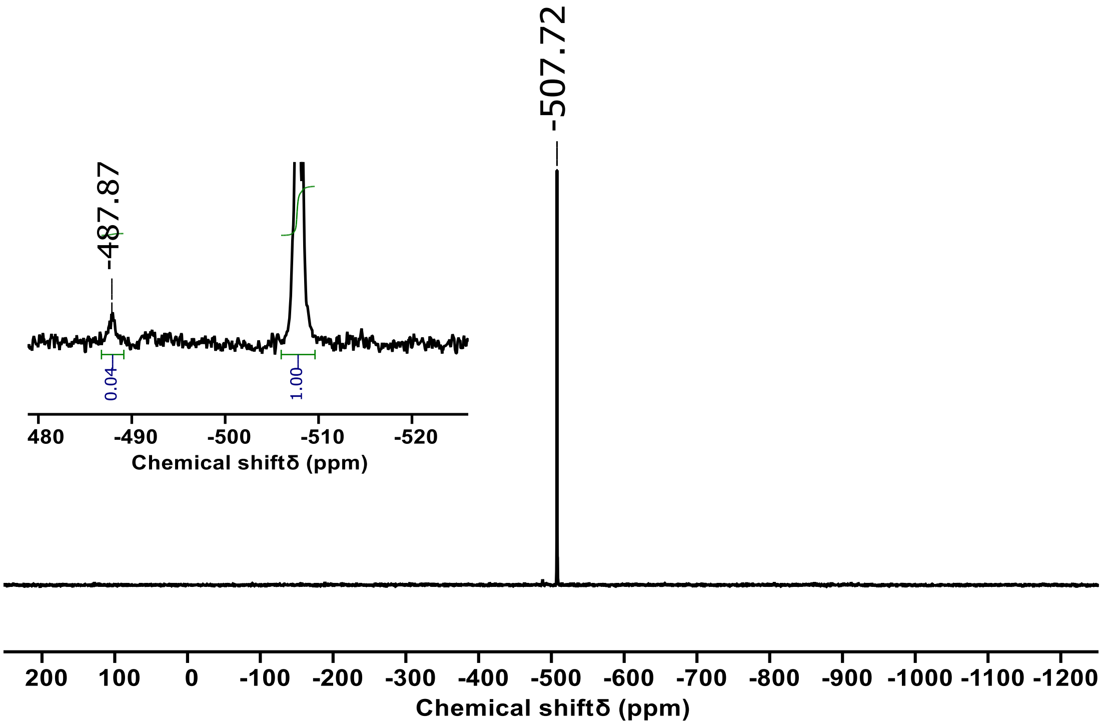


**Figure S3:** $^{51}V$ NMR spectrum (500 MHz) of $(TBA)_3[VW_5O_{19}]$ in $CD_3CN$.

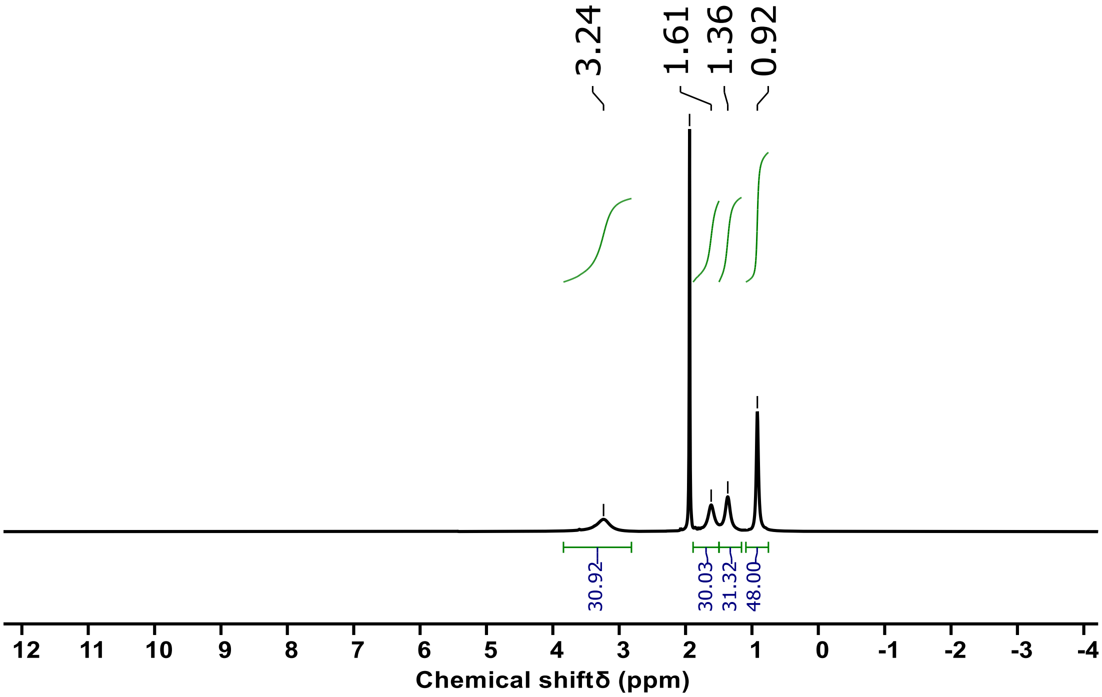


**Figure S4:** 1H NMR spectrum (500 MHz) of $(TBA)_4[VW_5O_{19}]$ in $CD_3CN$.

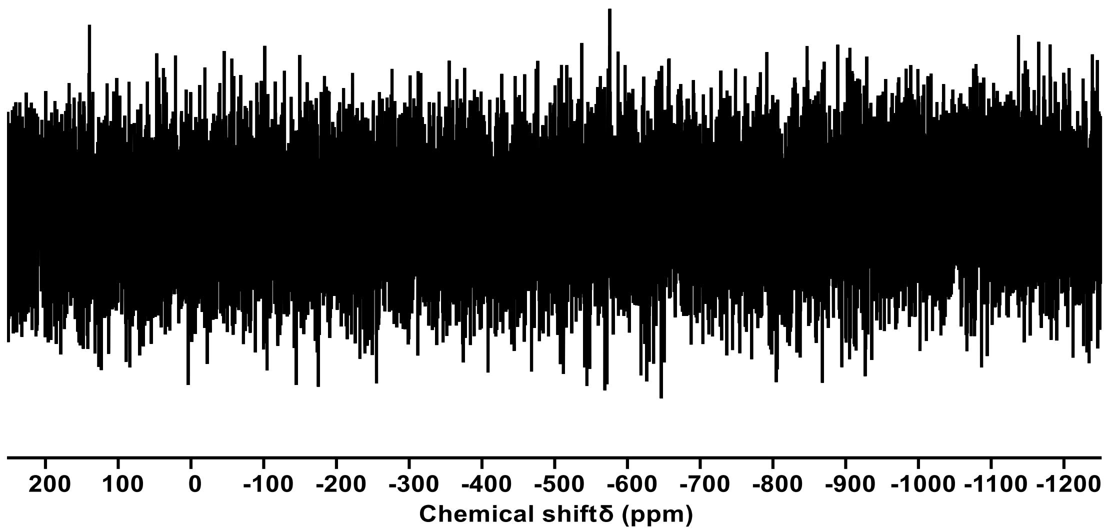


**Figure S5:** $^{51}V$ NMR spectrum (500 MHz) of $(TBA)_4[VW_5O_{19}]$ in $CD_3CN$.

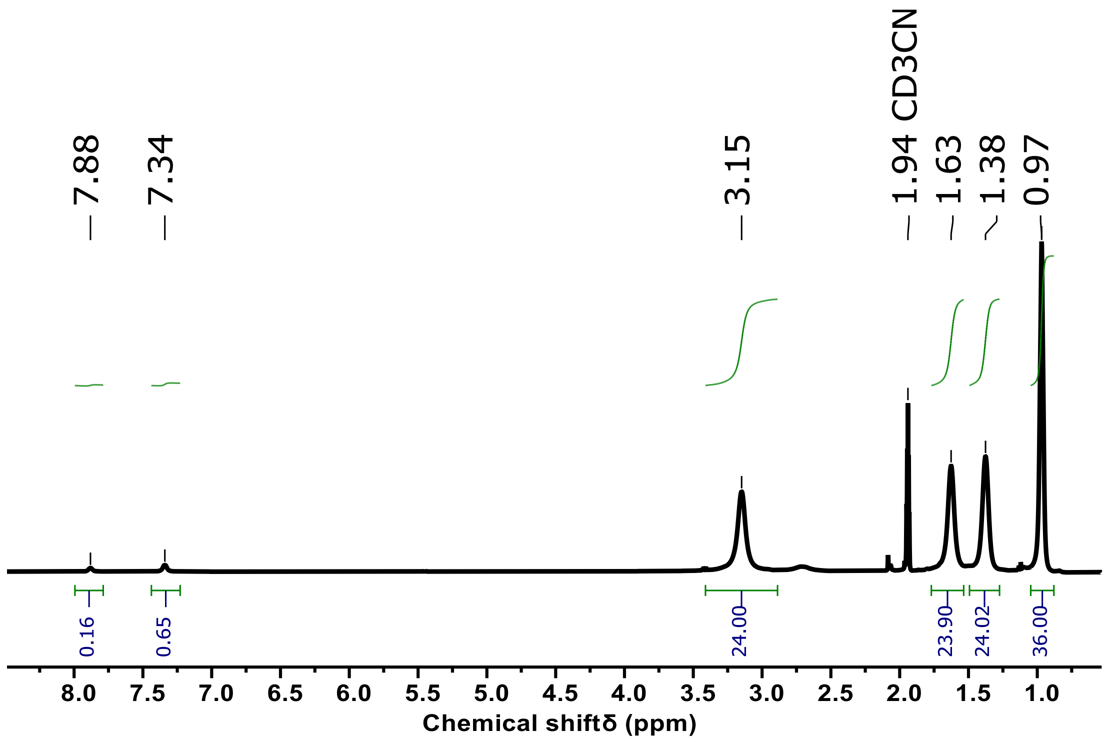


**Figure S6:** $^{1}H$ NMR spectrum (500 MHz) of $(TBA)_3[VW_5O_{19}H]$ in $CD_3CN$.

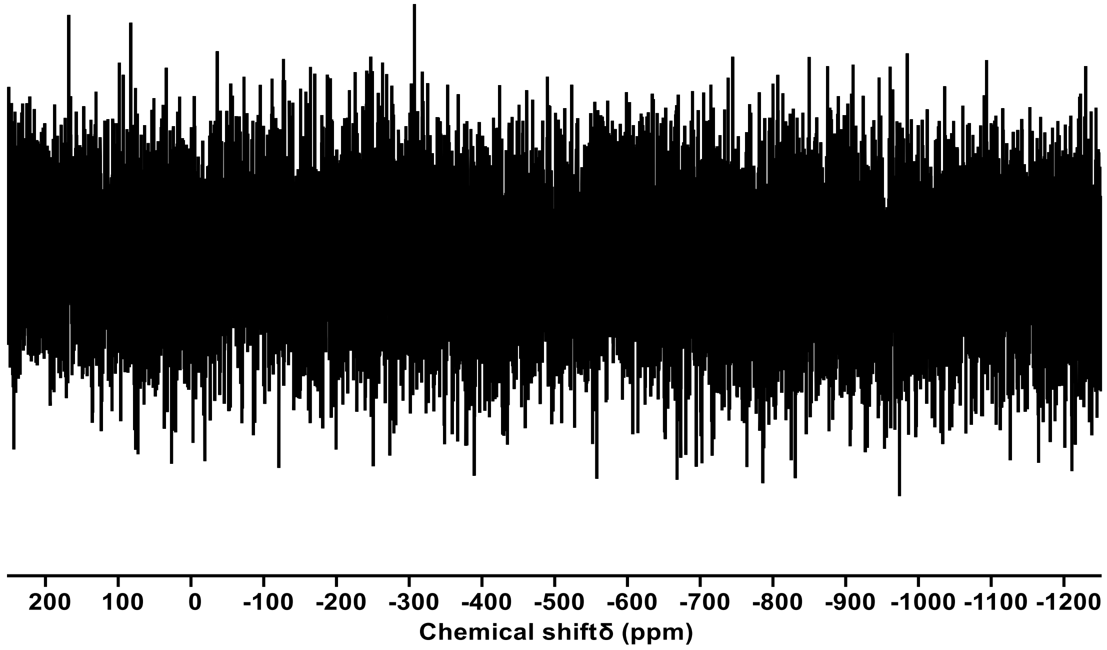


**Figure S7:** $^{51}V$ NMR spectrum (500 MHz) of $(TBA)_3[VW_5O_{19}H]$ in $CD_3CN$.

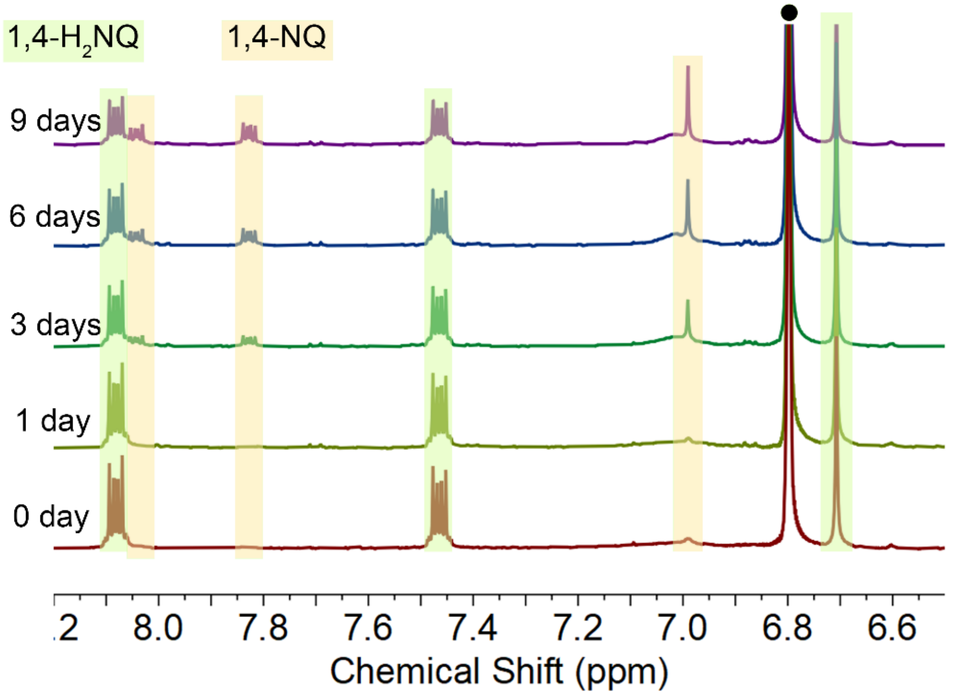


**Figure S8:** $^{1}H$-NMR spectra of 1,4-naphthohydroquinone (1,4-$H_2NQ$) after exposure to two equivalents of $\mathbf{[VW_5O_{19}]^{3-}}$ at 21°C for 9 days recorded in $CD_3CN$ (Trial A). Black circle represents the internal standard mesitylene.

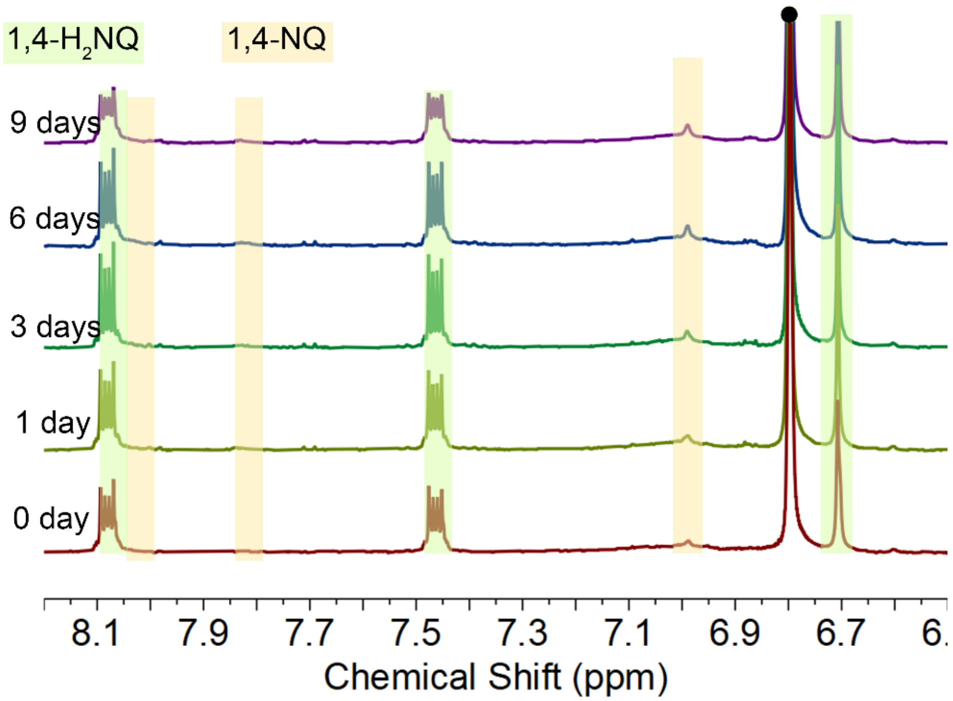


**Figure S9:** $^{1}$H-NMR spectra of 1,4-naphthohydroquinone (1,4-$H_2NQ$) after exposure to two equivalents of **$[VW_5O_{19}]^{3-}$** at 21°C for 9 days recorded in $CD_3CN$ (Trial B). Black circle represents the internal standard mesitylene.

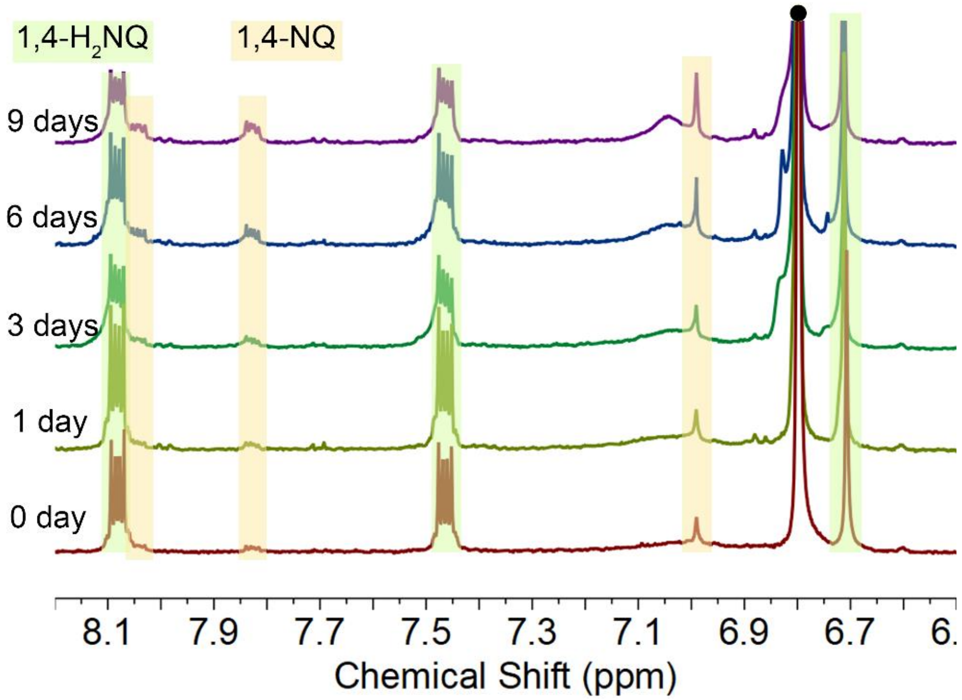


**Figure S10:** $^{1}$H-NMR spectra of 1,4-naphthohydroquinone (1,4-$H_2NQ$) after exposure to two equivalents of **$[VW_5O_{19}]^{3-}$** at 21°C for 9 days recorded in $CD_3CN$ (Trial C). Black circle represents the internal standard mesitylene.

**Table S1:** $BDFE_{adj}$ calculated from equilibrated reactions between **$[VW_5O_{19}]^{3-}$** and half equivalent of 1,4-$H_2$NQ described in **Figures S8-S10**, using the equation of $BDFE_{adj}$.

| | 1,4-$H_2$NQ | | 1,4-NQ | | log([1,4-$H_2$NQ]/[1,4-NQ])[a] | $BDFE_{adj}$ (kcal $mol^{-1}$) |
|---|---|---|---|---|---|---|
| | 8.09 ppm (2H) | 7.47 ppm (2H) | 8.05 ppm (2H) | 7.83 ppm (2H) | | |
| Trial | Integral | | | | | |
| A | 0.44 | 0.46 | 0.2 | 0.21 | 0.34 | 63.1 |
| B | 0.72 | 0.69 | 0.05 | 0.03 | 1.25 | 62.5 |
| C | 0.64 | 0.65 | 0.16 | 0.17 | 0.59 | 62.9 |
| | | | | | Avg. | 62.8 |
| | | | | | Std. Dev | 0.3 |

[a]: Conc. relative to the internal standard mesitylene.

Note: the BDFE(O-H) of 1,4-$H_2$NQ in MeCN (63.3 kcal mol-1) has not been reported; only the BDFE(O-H) in THF (62.6 kcal $mol^{-1}$) is reported in [8]. The value in MeCN is estimated by following the literature [9,10].

## 2. Infrared spectra

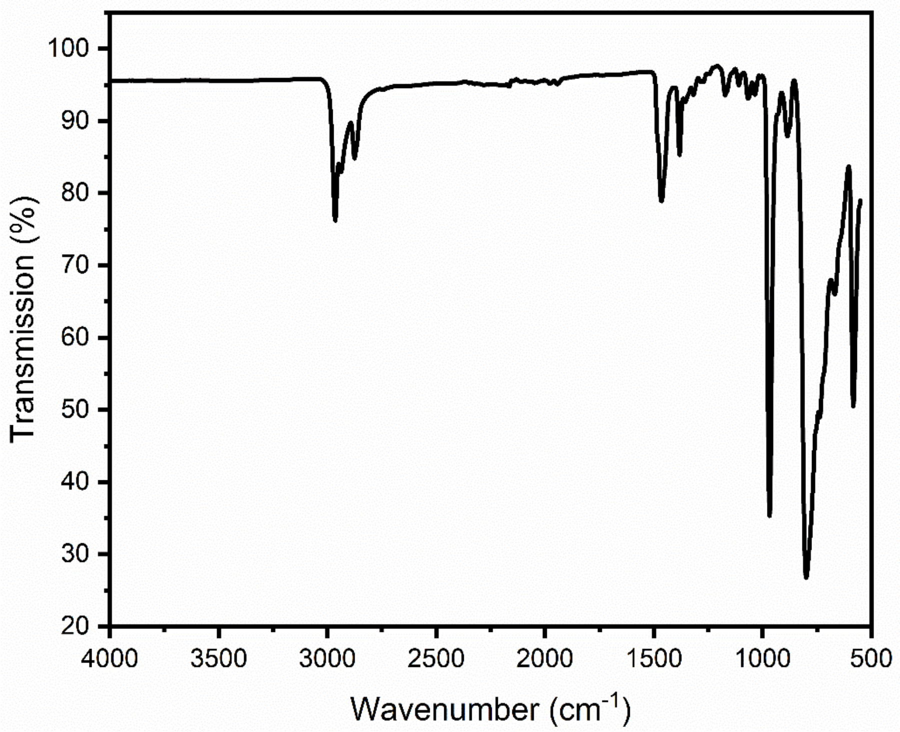


**Figure S11:** Infrared spectrum of $(TBA)_2[W_6O_{19}]$.

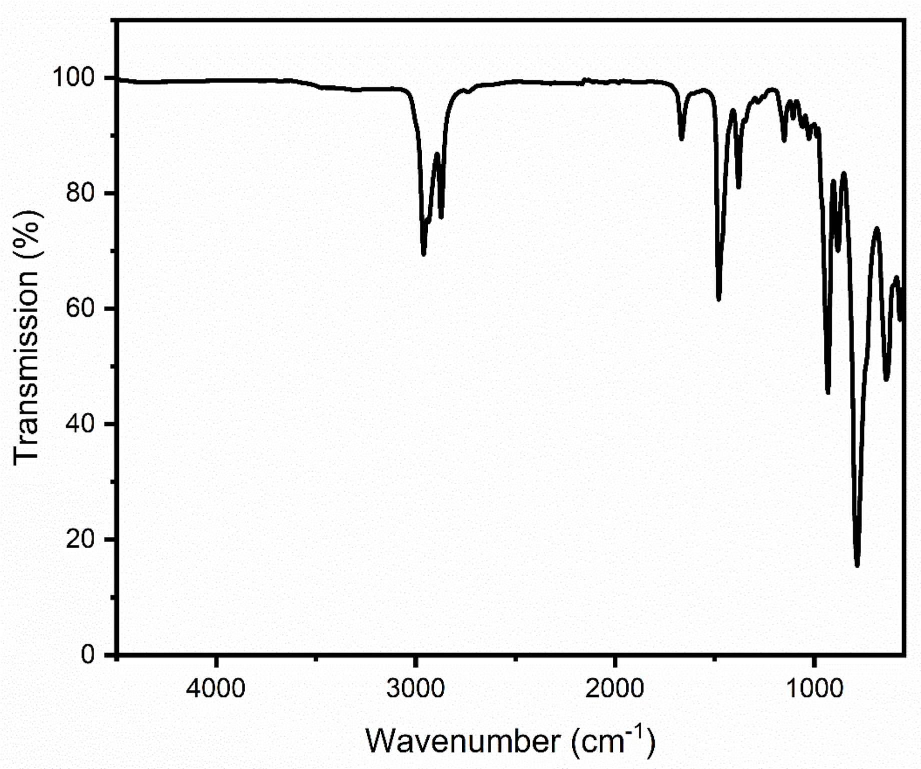


**Figure S12:** Infrared spectrum of $(TBA)_3[VW_5O_{19}]$.

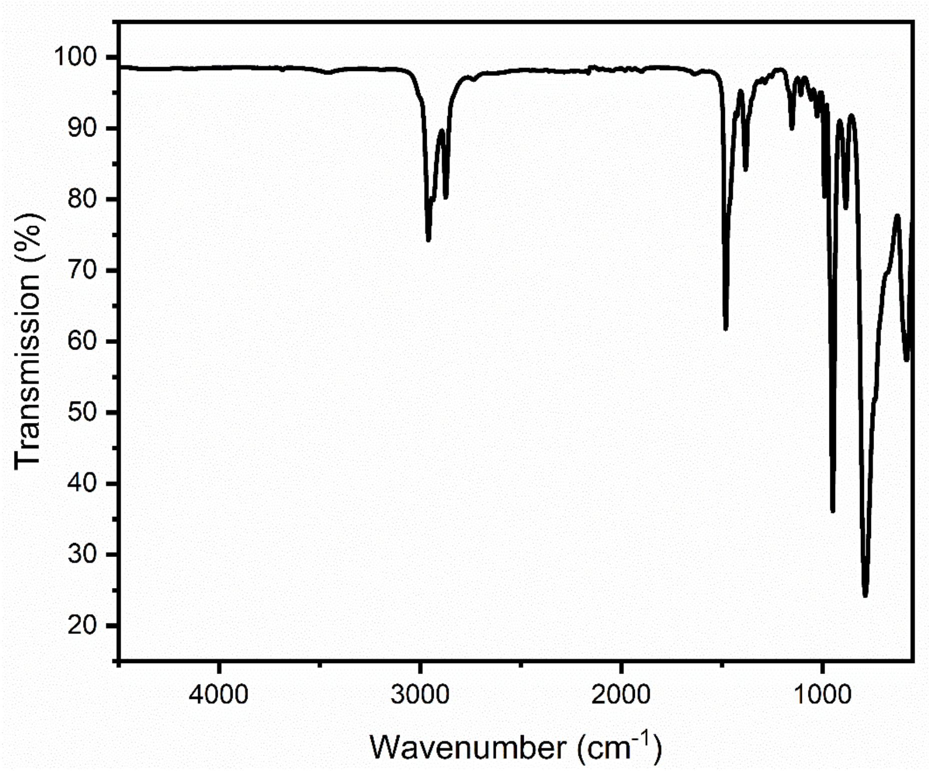


**Figure S13:** Infrared spectrum of $(TBA)_4[VW_5O_{19}]$.

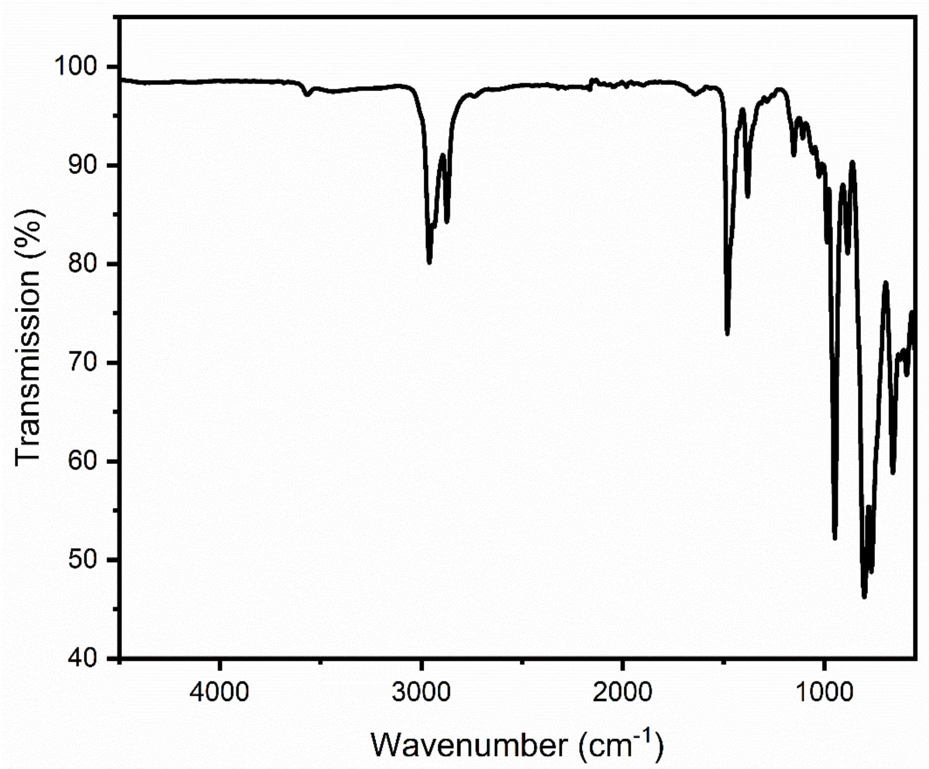


**Figure S14:** Infrared spectrum of $(TBA)_3[VW_5O_{19}H]$.

## 3. Electrochemistry

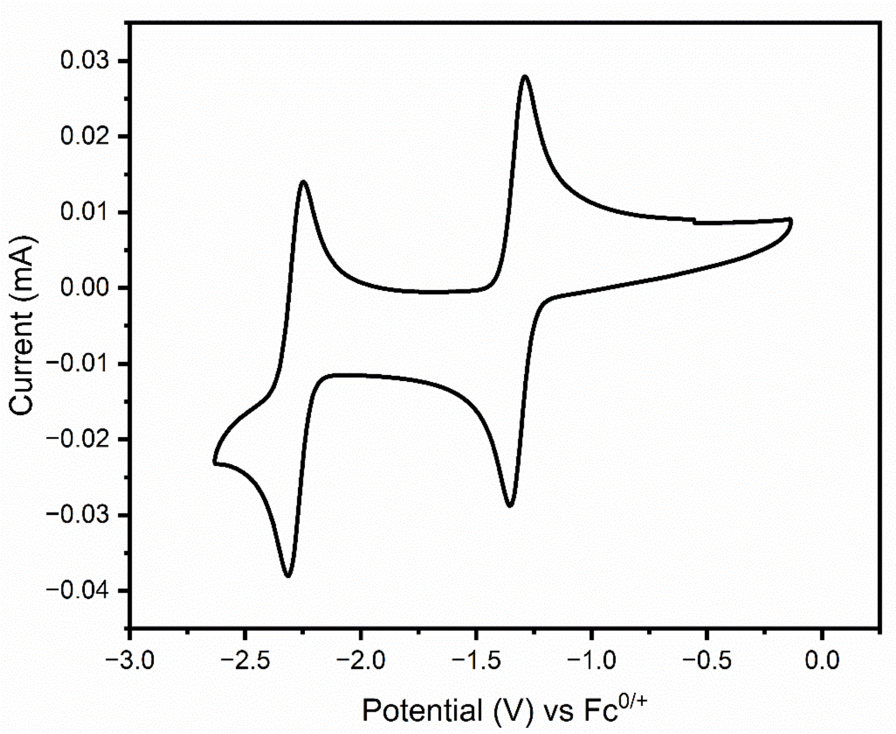


**Figure S15:** Cyclic voltammogram of $(TBA)_2[W_6O_{19}]$ (1 mM) in MeCN/TBA($PF_6$) (0.1 M). OCP −0.56 V.

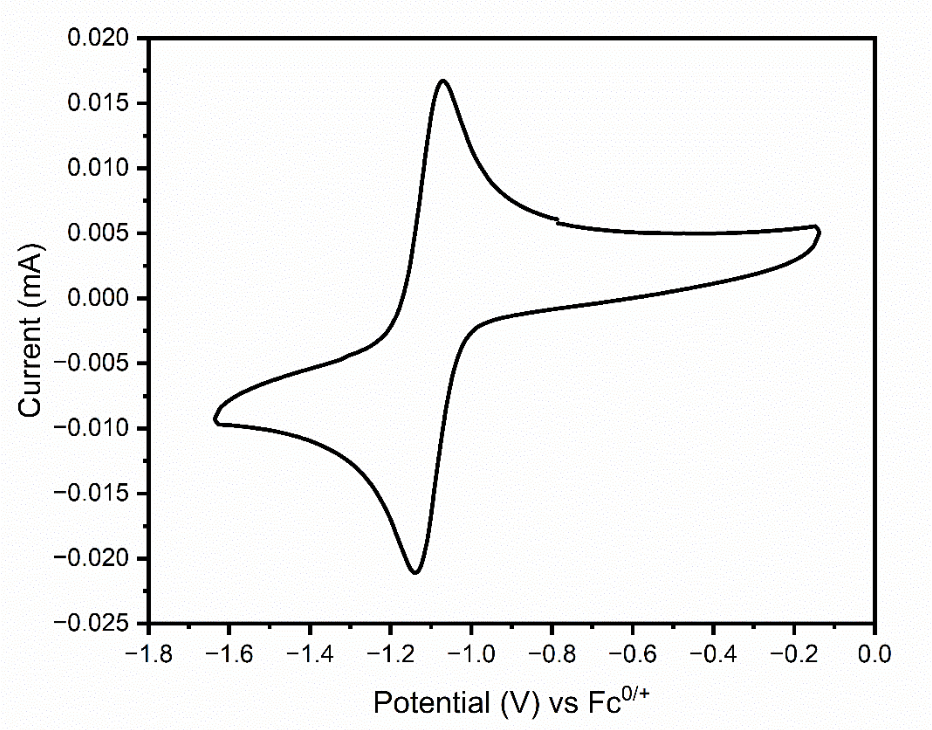


**Figure S16:** Cyclic voltammogram of $(TBA)_3[VW_5O_{19}]$ (1 mM) in MeCN/TBA($PF_6$) (0.1 M). OCP −0.78 V.

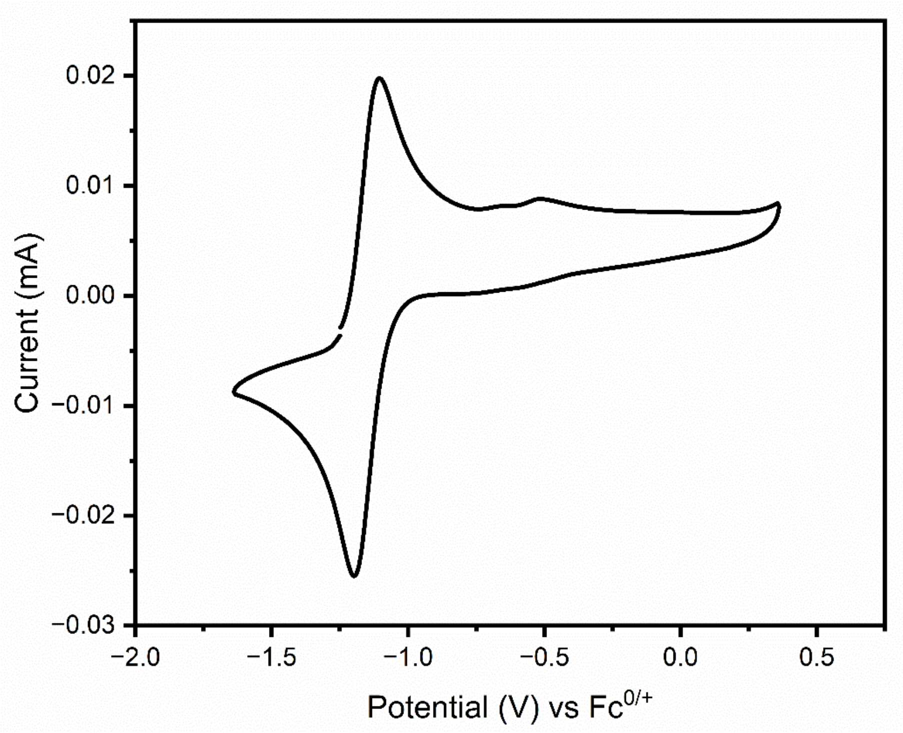


**Figure S17:** Cyclic voltammogram of $(TBA)_4[VW_5O_{19}]$ (1 mM) in MeCN/TBA($PF_6$) (0.1 M). OCP −1.25 V.

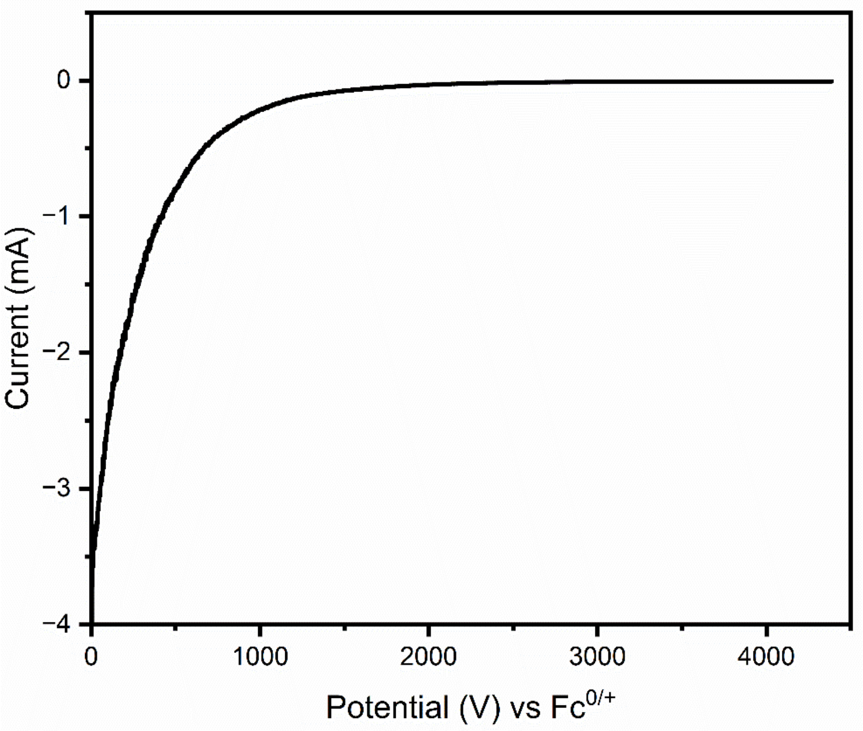


**Figure S18:** Chronoamperometry of $(TBA)_2[W_6O_{19}]$ (1 mM) in MeCN/TBA($PF_6$) (0.1 M) at −1.64 V.

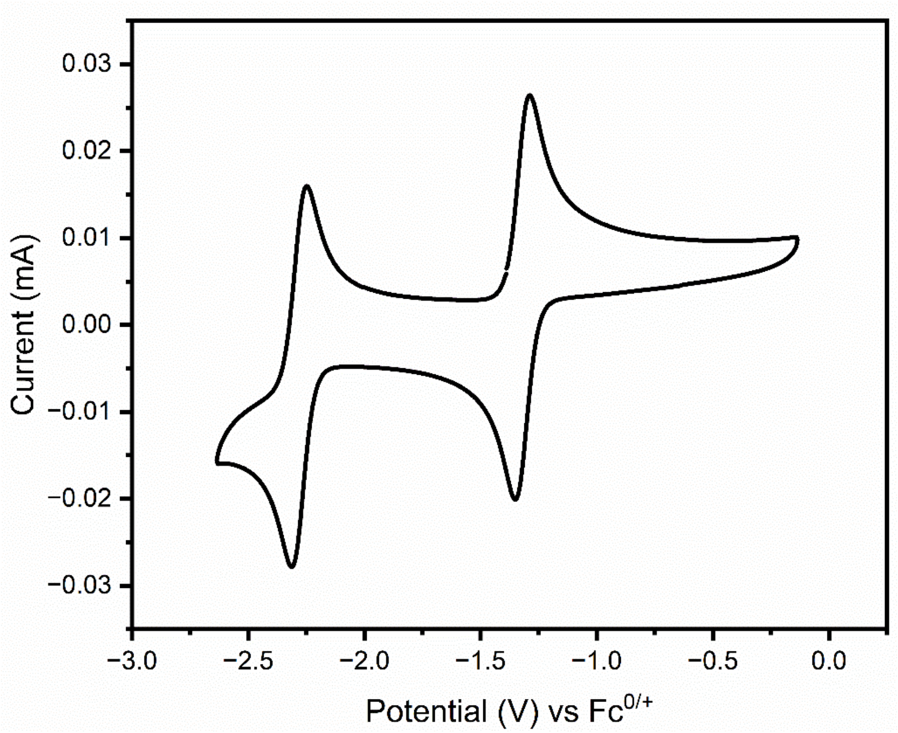


**Figure S19:** Post CA CV of $(TBA)_2[W_6O_{19}]$ (1 mM) in MeCN/TBA($PF_6$) (0.1 M). OCP −1.39 V.

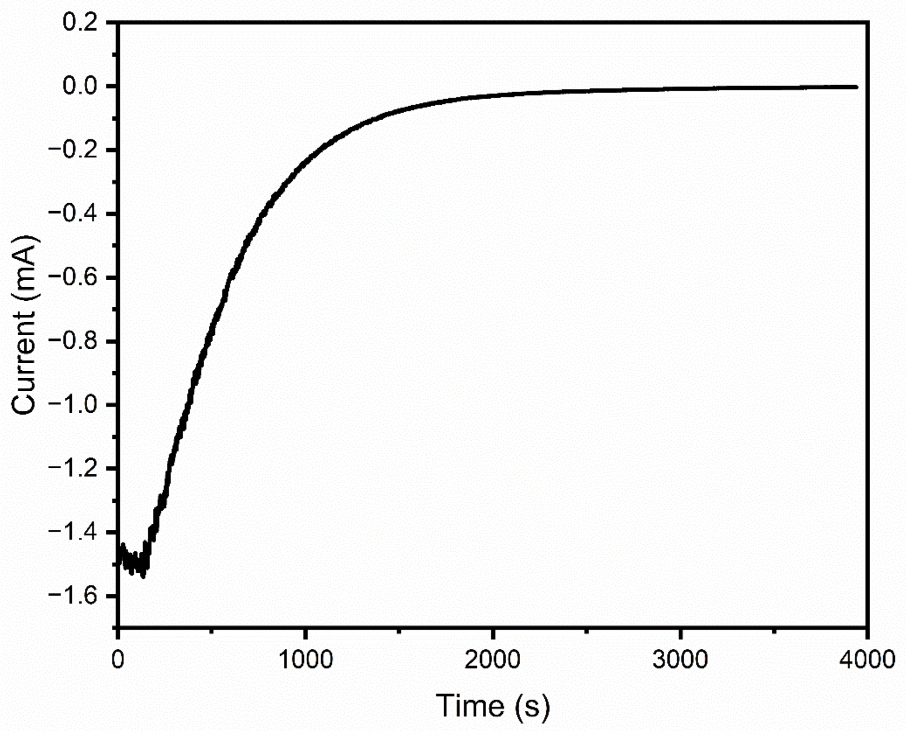


**Figure S20:** Chronoamperometry of $(TBA)_3[VW_5O_{19}]$ (1 mM) in MeCN/TBA($PF_6$) (0.1 M) at −1.39 V.

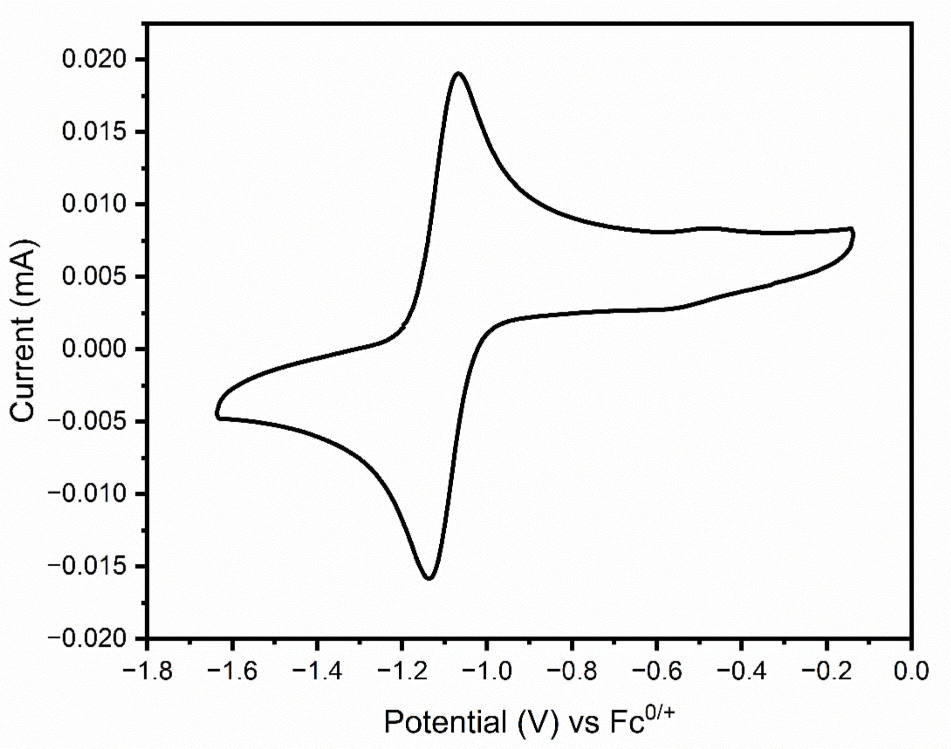


**Figure S21:** Post CA CV of (TBA)[$VW_5O_{19}$] (1 mM) in MeCN/TBA($PF_6$) (0.1 M). OCP −1.20 V.

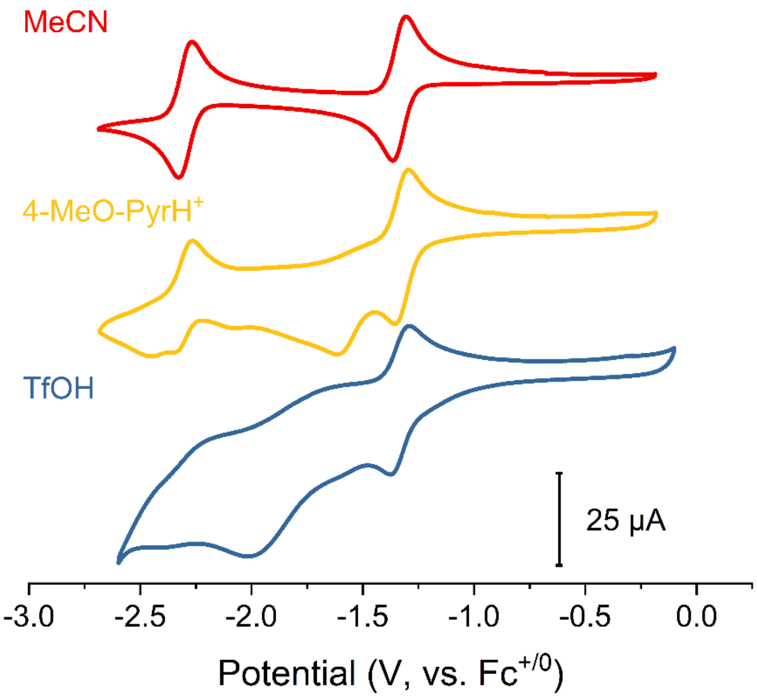


**Figure S22:** Cyclic voltammograms of 1 mM $\mathbf{[W_6O_{19}]^{2-}}$ obtained in acetonitrile in the presence of 2 mM various organic acids with the scan rate of 100 mV/s, using 0.1 M TBA($PF_6$) as the supporting electrolyte. Ferrocene is used for each measurement as the internal standard. The corresponding acids are listed in **Table S2**.

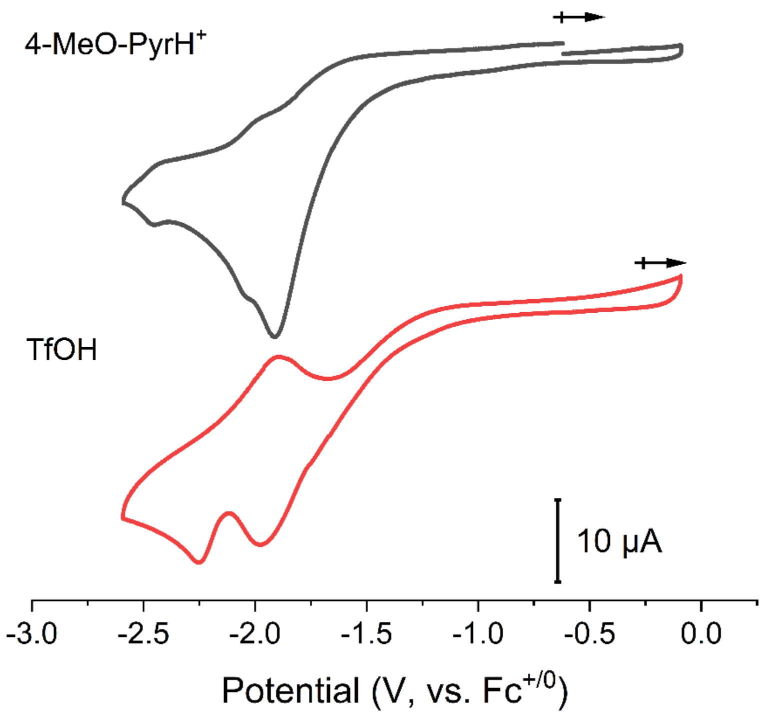


**Figure S23.** Cyclic voltammograms of 2 mM (upper) 4-MeO-PyrHBF$_4$ and (bottom) TfOH in acetonitrile with the scan rate of 100 mV/s, using 0.1 M [$^n$Bu$_4$N]PF$_6$ as the supporting electrolyte.

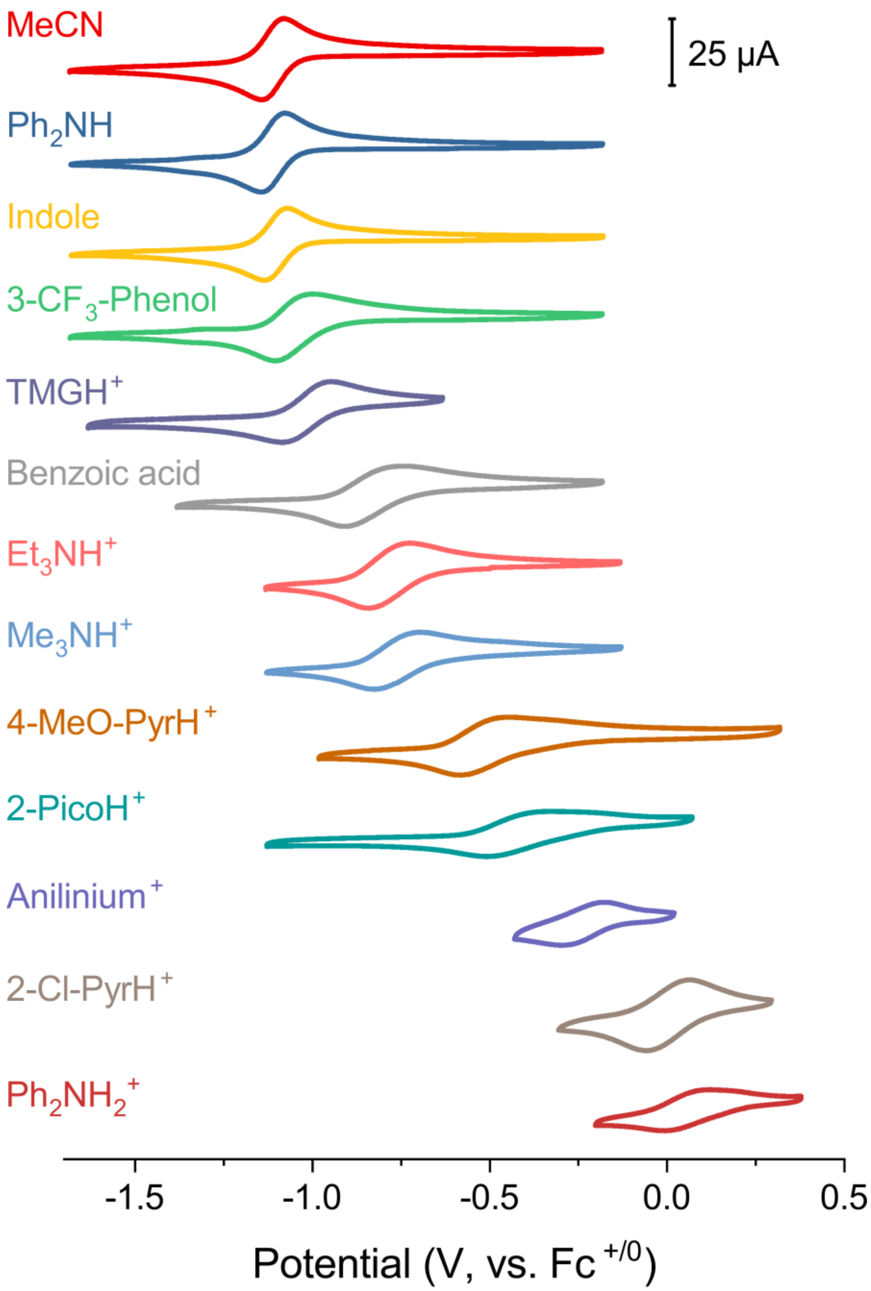


**Figure S24:** Cyclic voltammograms of 1 mM $\mathbf{[VW_5O_{19}]^{3-}}$ obtained in acetonitrile in the presence of 2 mM various organic acids with the scan rate of 100 mV/s, using 0.1 M TBA($PF_6$) as the supporting electrolyte. Ferrocene is used for each measurement as the internal standard. The corresponding acids are listed in **Table S2**.

**Table S2:** pKa values of various organic acids in acetonitrile.

| Acid | Abbreviation | p$K_a$(MeCN) | Ref. |
|---|---|---|---|
| Acetonitrile | MeCN | 39.5 | [1,2] |
| Diphenylamine | $Ph_2NH$ | 34.3 | [1,3] |
| Indole | | 32.57 | [4] |
| 3-Trifluoromethyl-Phenol | 3-$CF_3$-phenol | 26.5 | [4] |
| 1,1,3,3-Tetramethylguanidinium tetrafluoroborate | $TMGH^+$ | 23.35 | [5] |
| Benzoic acid | | 21.5 | [6] |
| Triethylammonium tetrafluoroborate | $Et_3NH^+$ | 18.83 | [5] |
| Trimethylammonium chloride | $Me_3NH^+$ | 17.61 | [5] |
| 4-Methoxyl-Pyridium tetrafluoroborate | 4-MeO-$PyrH^+$ | 14.24 | [5] |
| 2-Picodinium tetrafluoroborate | 2-$PicoH^+$ | 13.28 | [5] |
| Anilinium tetrafluoroborate | $Anilinium^+$ | 10.64 | [5] |
| 2-Chloro-Pyridium tetrafluoroborate | 2-Cl-$PyrH^+$ | 6.79 | [5] |
| Diphenylammonium tetrafluoroborate | $Ph_2NH_2^+$ | 5.98 | [5] |
| Trifluoromethanesulfonic acid | TfOH | 0.7 | [7] |

**Table S3:** Potential of the V(V)/V(IV) redox couple of $(TBA)_3[VW_5O_{19}]$ (1 mM) in the presence of 2 eq. of organic acids (in MeCN with 0.1 M TBA($PF_6$).

| Acid | P$k_a$(MeCN) | Reduction potential (V) (vs $Fc^{0/+}$) |
|---|---|---|
| Ph2NH | 34.3 | -1.111 |
| Indole | 32.57 | -1.102 |
| 7-NO2 indole | 29.99 | -1.109 |
| 3-CF3 phenol | 26.5 | -1.050 |
| Benzonic acid | 21.5 | -0.826 |
| 4-MeO-PyH | 14.24 | -0.512 |
| AnilineH[BF4] | 10.64 | -0.205 |
| 2-Cl-PyrH[BF4] | 6.79 | 0.034 |
| Ph2NH2[BF4] | 5.98 | 0.054 |

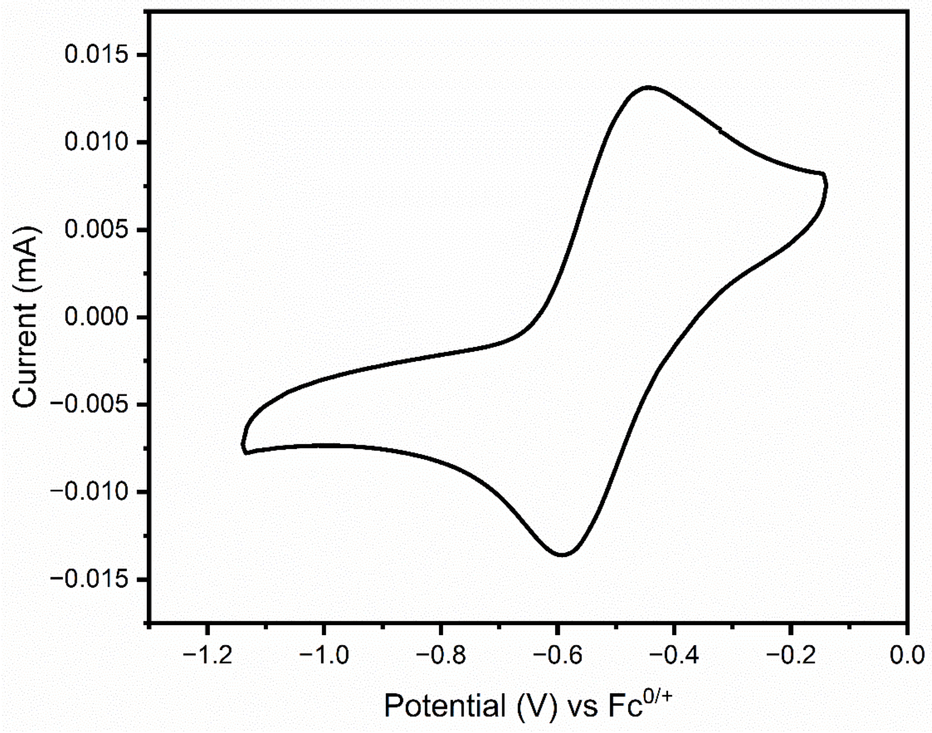


**Figure S25:** Pre bulk electrolysis CV of $(TBA)_3[VW_5O_{19}]$ (1 mM) in MeCN/TBA($PF_6$) (0.1 M) in presence of 2 eq. 2,6-lutidininium tetrafluoroborate. OCP −0.32 V.

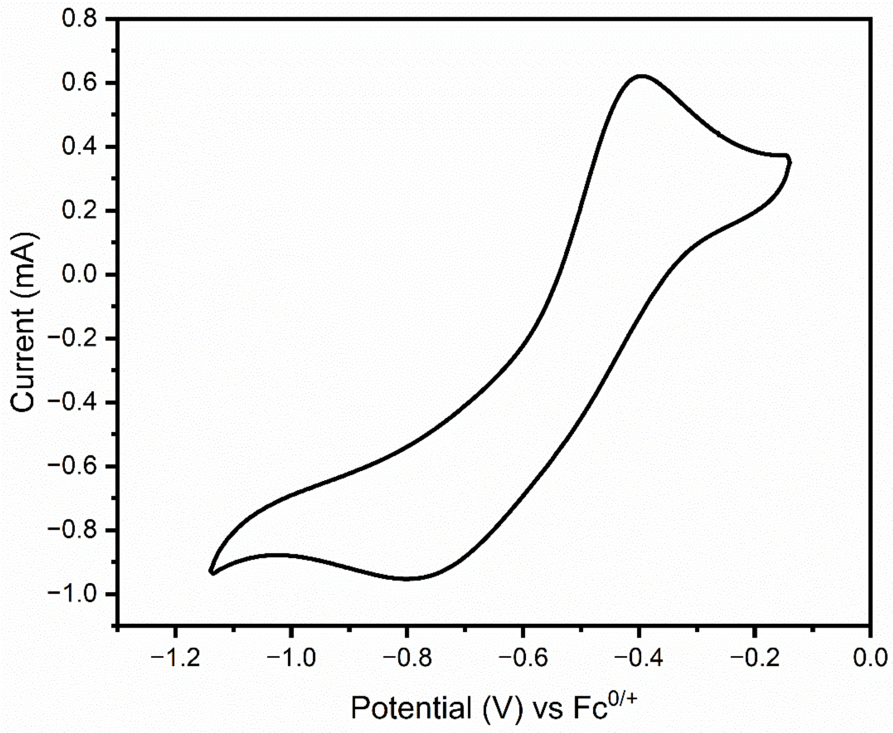


**Figure S26:** Pre bulk electrolysis CV of $(TBA)_3[VW_5O_{19}]$ (1 mM) in MeCN/TBA($PF_6$) (0.1 M) in presence of 2 eq. 2,6-lutidininium tetrafluoroborate on a Pt mesh working electrode. OCP −0.26 V.

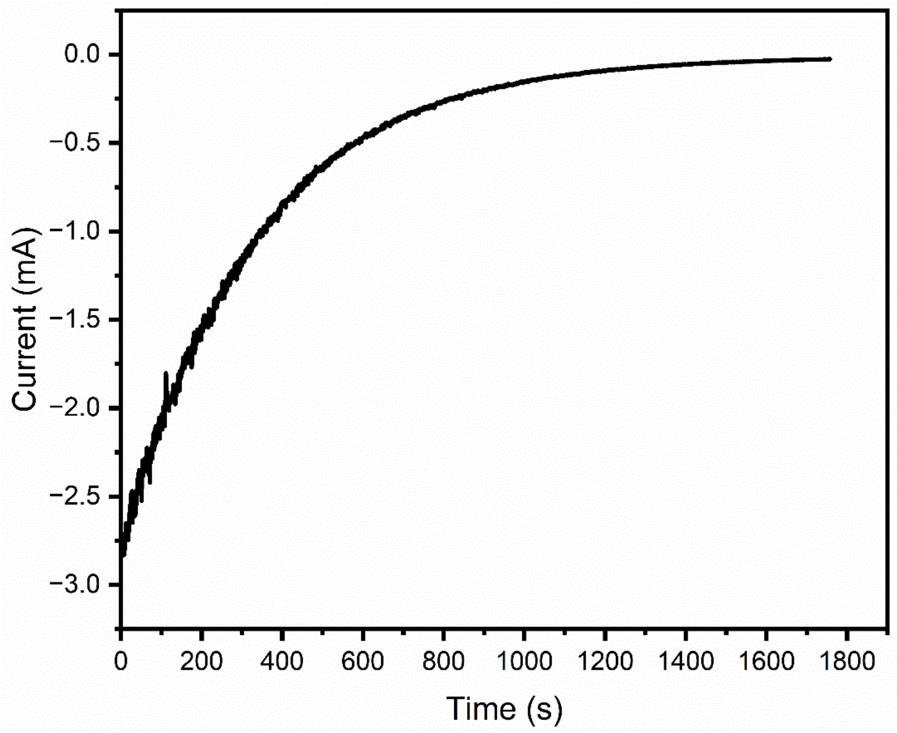


**Figure S27:** Chronoamperometry of $(TBA)_3[VW_5O_{19}]$ (1 mM) in MeCN/TBA($PF_6$) (0.1 M) at −1.04 V) in presence of 2 eq. 2,6-lutidininium tetrafluoroborate.

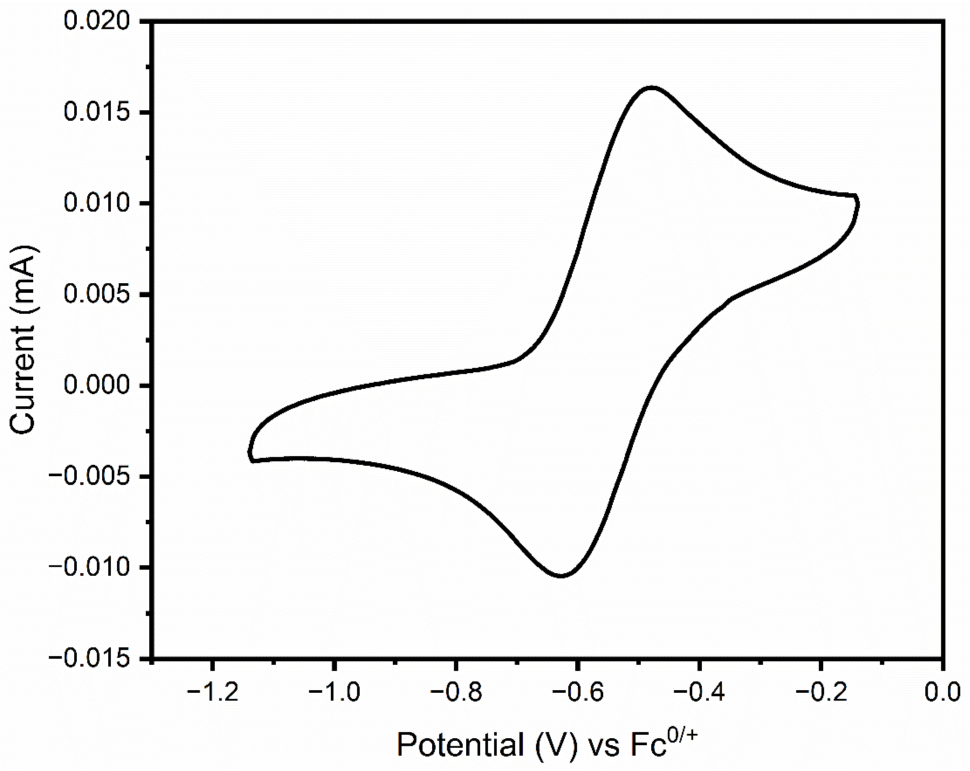


**Figure S28:** Post bulk electrolysis CV of $(TBA)_3[VW_5O_{19}]$ (1 mM) in MeCN/TBA($PF_6$) (0.1 M) in presence of 2 eq. 2,6-lutidininium tetrafluoroborate. OCP −0.71 V.

## 5. Electronic absorption spectroscopy

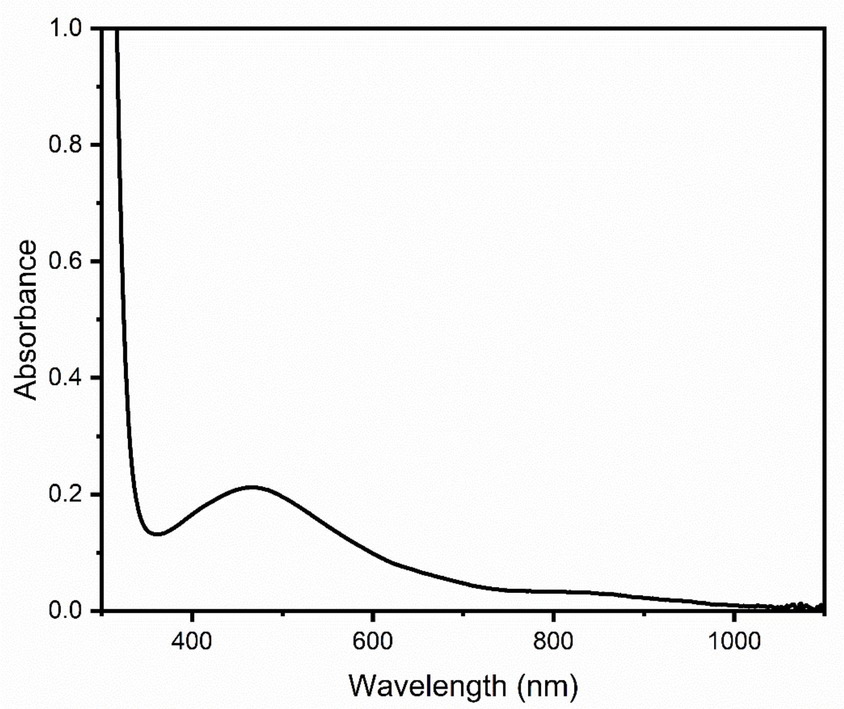


**Figure S29:** UV-Vis spectrum after bulk electrolysis of $(TBA)_3[VW_5O_{19}]$ (1 mM) in MeCN/TBA($PF_6$) (0.1 M) at −1.39 V followed by the addition of 2 eq. of 2,6-lutidininium tetrafluoroborate.

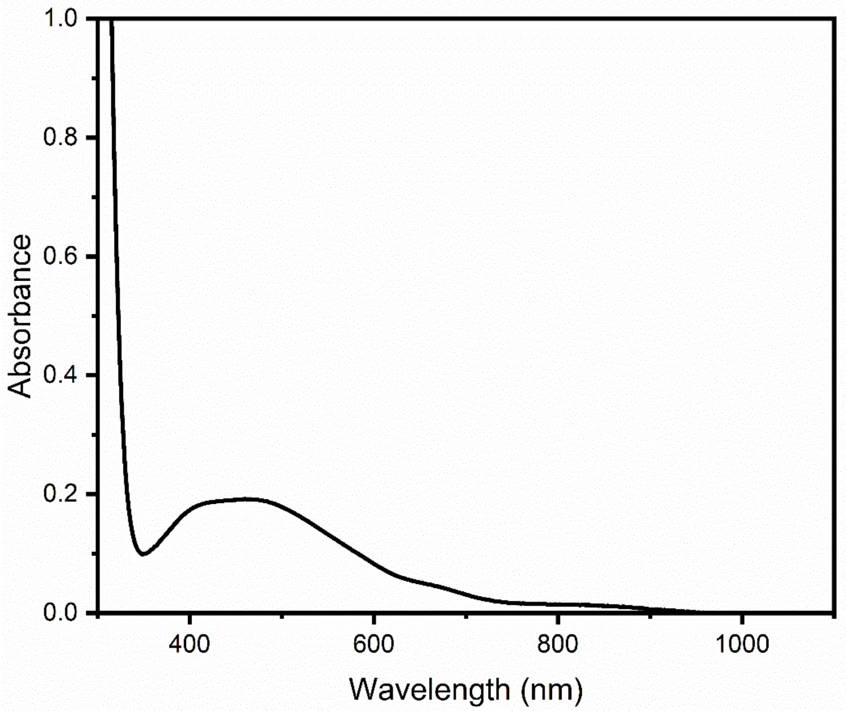


**Figure S30:** UV-Vis spectrum obtained after bulk electrolysis of $(TBA)_3[VW_5O_{19}]$ (1 mM) in MeCN/TBA($PF_6$) (0.1 M) in the presence of 2 eq. of 2,6-lutidininium tetrafluoroborate.

## 6. Single crystal X-ray diffraction data

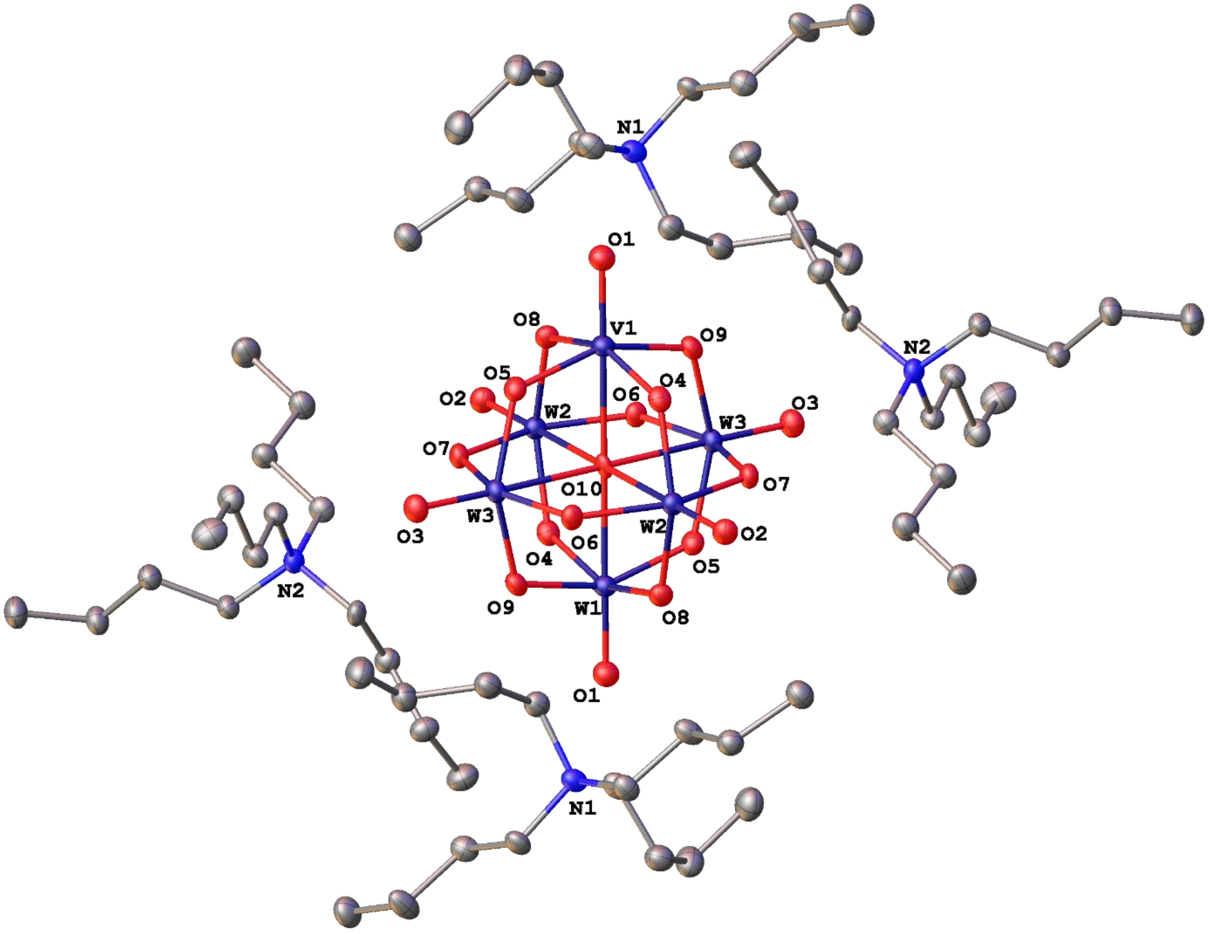


**Figure S31:** SCXRD structure of $(TBA)_4[VW_5O_{19}]$ obtained by vapor diffusion of diethyl ether into a saturated solution of the crude compound in MeCN. A complete Lindqvist unit and four TBA cations are shown for clarity though the asymmetric unit actually contains one half Lindqvist unit and two TBA cations in general positions (with one MeCN molecule). As discussed in the manuscript, the vanadium is disordered over all metal positions and therefore the labels given in this graphic are arbitrary.

**Table S4:** Crystal data and structure refinement for $(TBA)_4[VW_5O_{19}]$.

| | | |
|---|---|---|
| Empirical formula | C68 H150 N6 O19 V W5 | |
| Formula weight | 2326.12 | |
| Temperature | 100.00(10) K | |
| Wavelength | 1.54184 Å | |
| Crystal system | orthorhombic | |
| Space group | *Pbca* | |
| Unit cell dimensions | *a* = 17.68460(10) Å | a = 90° |
| | *b* = 16.95360(10) Å | b = 90° |
| | *c* = 28.29200(10) Å | g = 90° |
| Volume | 8482.44(8) $Å^3$ | |
| *Z* | 4 | |
| Density (calculated) | 1.821 $Mg/m^3$ | |
| Absorption coefficient | 13.582 $mm^{-1}$ | |
| *F*(000) | 4580 | |
| Crystal color, morphology | orange-brown, block | |
| Crystal size | 0.292 x 0.156 x 0.071 $mm^3$ | |
| Theta range for data collection | 3.936 to 80.264° | |
| Index ranges | -22 £ *h* £ 17, -21 £ *k* £ 20, -35 £ *l* £ 36 | |
| Reflections collected | 77921 | |
| Independent reflections | 9172 [*R*(int) = 0.0483] | |
| Observed reflections | 8955 | |
| Completeness to theta = 74.504° | 100.0% | |
| Absorption correction | Multi-scan | |
| Max. and min. transmission | 1.00000 and 0.27624 | |
| Refinement method | Full-matrix least-squares on $F^2$ | |
| Data / restraints / parameters | 9172 / 1 / 460 | |
| Goodness-of-fit on $F^2$ | 1.113 | |
| Final *R* indices [*I*>2sigma(*I*)] | *R*1 = 0.0350, *wR*2 = 0.0920 | |
| *R* indices (all data) | *R*1 = 0.0360, *wR*2 = 0.0927 | |
| Largest diff. peak and hole | 0.865 and -1.436 e.$Å^{-3}$ | |

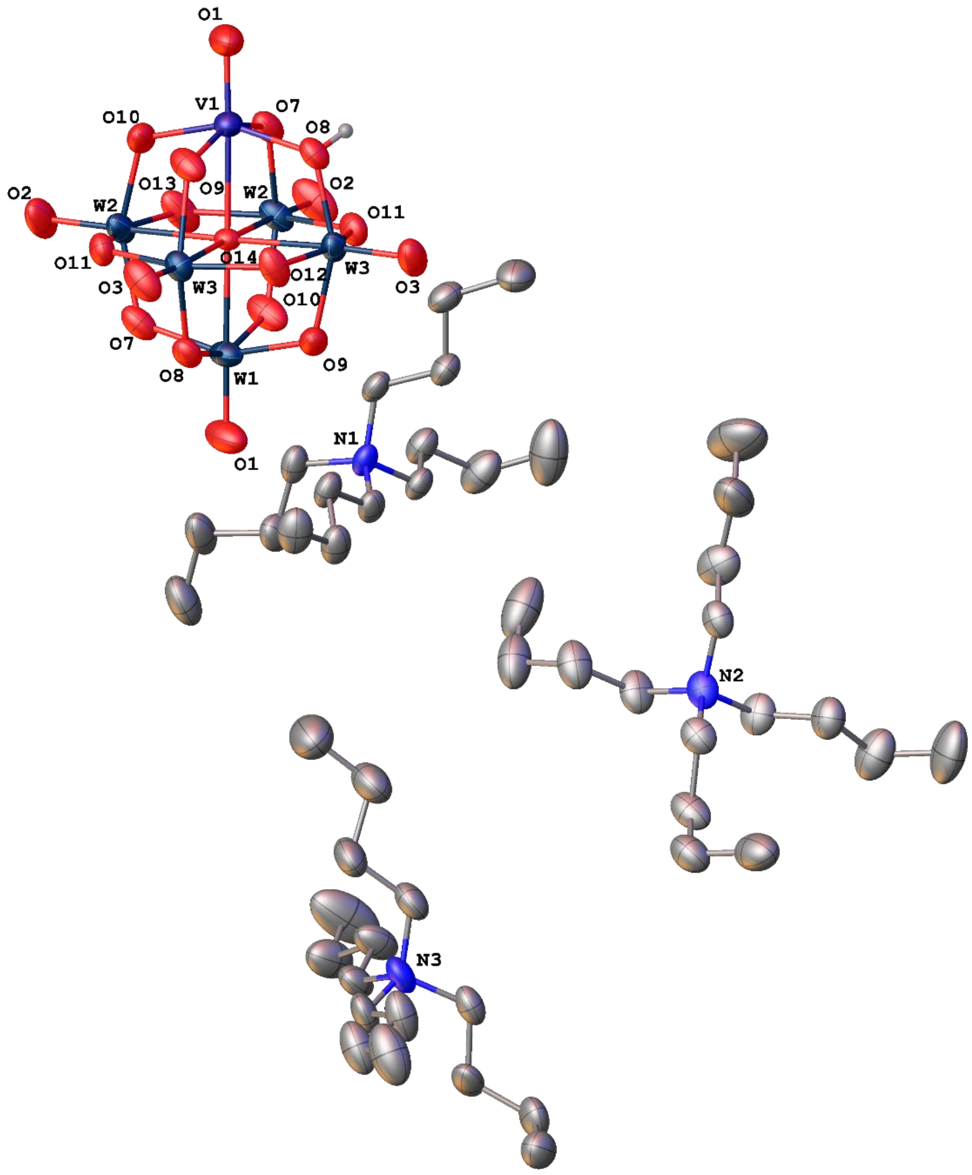


**Figure S32:** SCXRD structure of $(TBA)_3[VW_5O_{18}(OH)]$ obtained by vapor diffusion of diethyl ether into a concentrated solution of the $(TBA)_4[VW_5O_{19}]$ in MeCN in the presence of 2,6-lutidinium tetrafluoroborate. A complete Lindqvist unit and three TBA cations are shown for clarity though the asymmetric unit actually contains one-half of one cluster on a crystallographic two-fold axis, one-half of a second cluster on a crystallographic inversion center, and three tetrabutylammonium cations in general positions. As discussed in the manuscript, the vanadium is disordered over all metal positions and therefore the labels given in this graphic are arbitrary. Just as the two metal types (V and W) are disordered over all metal sites, the proton is likely disordered over multiple sites, perhaps even all 12 per cluster (due to the V/W disorder). Therefore, each O-H hydrogen atom (one per cluster) was placed on a single bridging O atom that had relatively shorter M-O distances and that minimized any potential intermolecular H...H contacts. Because both independent clusters in this structure lie on crystallographic symmetry elements, the occupancies of these hydrogen atoms were fixed at 0.50.

**Table S5:** Crystal data and structure refinement for $(TBA)_3[VW_5O_{18}(OH)]$

| | | |
|---|---|---|
| Empirical formula | C48 H109 N3 O19 V W5 | |
| Formula weight | 2002.57 | |
| Temperature | 100.00(10) K | |
| Wavelength | 1.54184 Å | |
| Crystal system | monoclinic | |
| Space group | *I*2/*a* | |
| Unit cell dimensions | *a* = 31.2939(3) Å | a = 90° |
| | *b* = 18.46294(11) Å | b = 119.9133(11)° |
| | *c* = 27.0952(2) Å | g = 90° |
| Volume | 13569.4(2) $Å^3$ | |
| *Z* | 8 | |
| Density (calculated) | 1.960 $Mg/m^3$ | |
| Absorption coefficient | 16.843 $mm^{-1}$ | |
| *F*(000) | 7704 | |
| Crystal color, morphology | purple-brown, block | |
| Crystal size | 0.17 x 0.11 x 0.04 $mm^3$ | |
| Theta range for data collection | 2.895 to 80.095° | |
| Index ranges | -39 £ *h* £ 36, -23 £ *k* £ 23, -25 £ *l* £ 34 | |
| Reflections collected | 115462 | |
| Independent reflections | 14586 [*R*(int) = 0.0387] | |
| Observed reflections | 13673 | |
| Completeness to theta = 67.684° | 100.0% | |
| Absorption correction | Multi-scan | |
| Max. and min. transmission | 1.00000 and 0.41340 | |
| Refinement method | Full-matrix least-squares on $F^2$ | |
| Data / restraints / parameters | 14586 / 107 / 793 | |
| Goodness-of-fit on $F^2$ | 1.051 | |
| Final *R* indices [*I*>2sigma(*I*)] | *R*1 = 0.0339, *wR*2 = 0.0773 | |
| *R* indices (all data) | *R*1 = 0.0366, *wR*2 = 0.0786 | |
| Largest diff. peak and hole | 2.232 and -3.030 e.$Å^{-3}$ | |

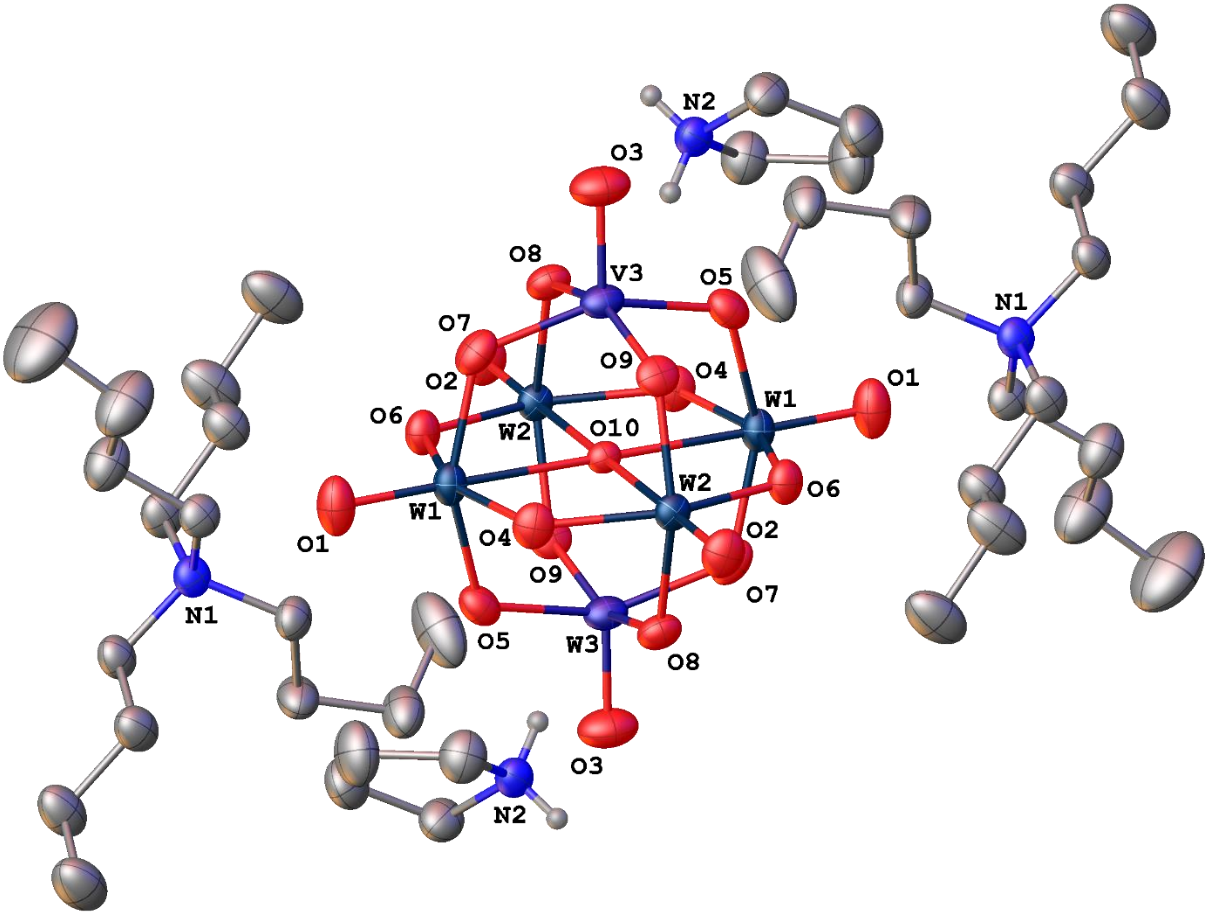


**Figure S33:** SCXRD structure of $(TBA)_2(PyrrH)_2[VW_5O_{19}]$ obtained by vapor diffusion of diethyl ether into a concentrated solution of the $(TBA)_4[VW_5O_{19}]$ in MeCN in the presence of pyrrolidinium tetrafluoroborate. A complete Lindqvist unit, two TBA cations, and two pyrrolidinium, cations are shown for clarity though the asymmetric unit actually contains one-half of a tetraanionic $VW_5$ cluster on a crystallographic inversion center that coincides with atom O10, one pyrrolidinium cation in a general position, and one tetrabutylammonium cation in a general position. The three unique metal sites are modeled as mixtures of the two atom types: W1:V1, 0.98:0.02, W2:V2, 0.79:0.21, and W3:V3, 0.73:0.27. The occupancies of the two metal types at each disordered site were constrained to add to one. The total W and V over the three disordered sites was constrained to be 2.5 and 0.5, respectively. Therefore, the labels given in this graphic are arbitrary. The innermost $-CH_2CH_2-$ linkage of the pyrrolidinium cation is modeled as disordered over two positions (0.67:0.33), though this is omitted here.

**Table S6:** Crystal data and structure refinement for $(TBA)_2(PyrrH)_2[VW_5O_{19}]$

| | | |
|---|---|---|
| Empirical formula | C40 H92 N4 O19 V W5 | |
| Formula weight | 1903.36 | |
| Temperature | 173.00(10) K | |
| Wavelength | 1.54184 Å | |
| Crystal system | monoclinic | |
| Space group | $P2_1/c$ | |
| Unit cell dimensions | *a* = 9.44620(10) Å | a = 90° |
| | *b* = 16.85360(10) Å | b = 97.3480(10)° |
| | *c* = 17.84050(10) Å | g = 90° |
| Volume | 2816.93(4) $Å^3$ | |
| *Z* | 2 | |
| Density (calculated) | 2.244 $Mg/m^3$ | |
| Absorption coefficient | 20.244 $mm^{-1}$ | |
| *F*(000) | 1810 | |
| Crystal color, morphology | red-violet, plate | |
| Crystal size | 0.172 x 0.11 x 0.029 $mm^3$ | |
| Theta range for data collection | 3.622 to 80.574° | |
| Index ranges | -11 £ *h* £ 11, -21 £ *k* £ 20, -22 £ *l* £ 22 | |
| Reflections collected | 48619 | |
| Independent reflections | 6086 [*R*(int) = 0.0527] | |
| Observed reflections | 5822 | |
| Completeness to theta = 74.504° | 100.0% | |
| Absorption correction | Multi-scan | |
| Max. and min. transmission | 1.00000 and 0.29113 | |
| Refinement method | Full-matrix least-squares on $F^2$ | |
| Data / restraints / parameters | 6086 / 92 / 406 | |
| Goodness-of-fit on $F^2$ | 1.060 | |
| Final *R* indices [*I*>2sigma(*I*)] | *R*1 = 0.0257, *wR*2 = 0.0628 | |
| *R* indices (all data) | *R*1 = 0.0271, *wR*2 = 0.0637 | |
| Extinction coefficient | 0.000246(12) | |
| Largest diff. peak and hole | 0.751 and -0.883 e.$Å^{-3}$ | |

## 7. EPR Spectra

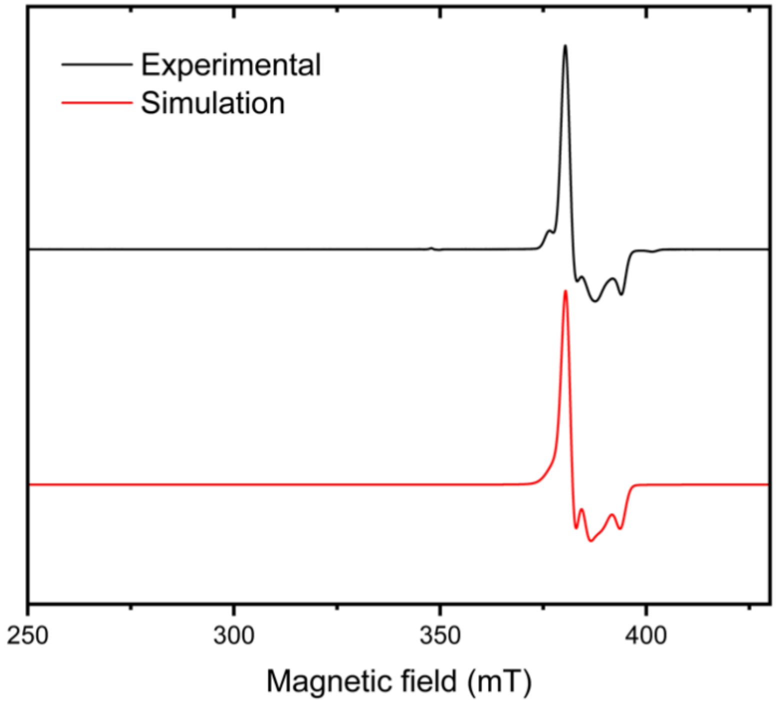


**Figure S34:** 10 K X-band EPR spectrum of reduced $[W_6O_{19}]^{2-}$ in MeCN solution containing 0.1 M TBA($PF_6$) (black line) and simulated spectrum (red line). The simulation coincides with a mixture of two species. One corresponds to previous reported parameters for $[W_6O_{19}]^{3-}$, characterized by $g_1$ = 1.759, $g_2$ = 1.702, A(W) = [0.371 0.177] MHz and lw (gaussian) = 2.27 mT. The second species considered impurity are characterized by the simulated parameters $g_1$ = 1.772, $g_2$ = 1.741, $g_3$ = 1.720, A(W) = [5.29 5.44 4.86] MHz.

## 8. Computational methods and calculations

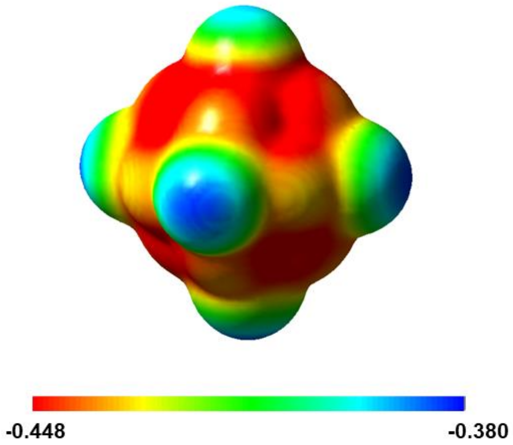


**Figure S35:** Molecular Electrostatic Potential (MEP) for **$[VW_5O_{19}]^{4-}$**. More nucleophilic sites are depicted in red, while more electrophilic sites are in blue.

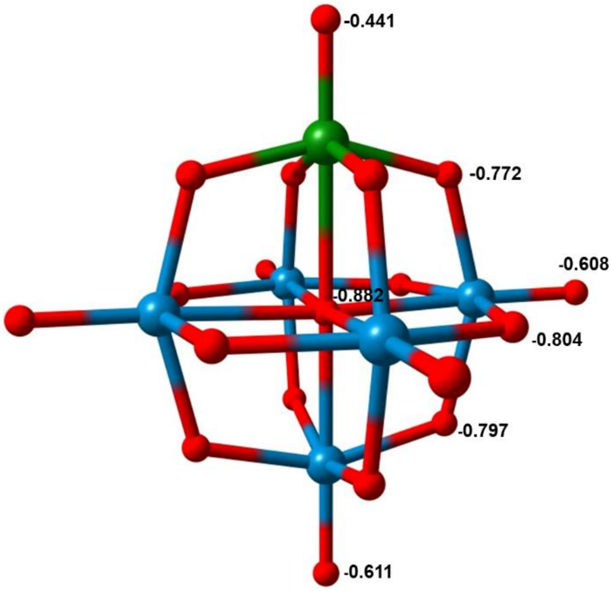


**Figure S36:** Calculated Mulliken charges for different types of oxygen for $\mathbf{[VW_5O_{19}]^{3-}}$.

**Table S7.** Effect of reduction and protonation on the high frequency vibrational frequencies ($cm^{-1}$) of $[VW_5O_{19}]^{3-}$, $[VW_5O_{19}]^{4-}$ and $[H_{v\text{-}o\text{-}w}VW_5O_{19}]^{3-}$.

| $[VW_5O_{19}]^{3-}$ | $[VW_5O_{19}]^{4-}$ | $[H_bVW_5O_{19}]^{3-}$ | **Assigments[1]** |
|---|---|---|---|
| 1066 | 1031 | 1050 | $V\text{-}O_t$ |
| 997 | 976 | 995, **987** | $M_{ax}\text{-}O_t$, $M_{eq}\text{-}O_t$ |
| 977, 972 | 955, 951 | **976**, 972, **971**, 968 | $M_{eq}\text{-}O_t$, $M_{ax}\text{-}O_t$ |
| 816 | 829 | **834**, 827 | $M_{ax}\text{-}O_b$ |
| 780 | 794 | 805, **802** | $M_{eq}\text{-}O_b$, $M_{ax}\text{-}O_b$ |
| 776 | 777 | 759 | $M_{ax}\text{-}O_b$ |

[1]axial (ax) refers to metals along the $C_4$ axis and equatorial (eq) to the metal atoms not on the $C_4$ axis for $[VW_5O_{19}]$ ion. The extra vibrational frequencies for $[H_bVW_5O_{19}]^{3-}$ are highlighted in bold and arise from the drop in symmetry from $C_{4V}$ to $C_s$.

**Table S8.** Calculated reduction potentials for $[W_6O_{19}]^{2-}$ and $[VW_5O_{19}]^{3-}$. Potentials are given in V vs. Fc/Fc$^+$

| | $[W_6O_{19}]^{2-}$ | $[VW_5O_{19}]^{3-}$ |
|---|---|---|
| $E_1$ | -1.51 | -1.05 |
| $E_2$ | -2.44 | -2.96 |

**Table S9:** XYZ coordinates for **$[W_6O_{19}]^{2-}$**.

W -0.209621000 -0.111679000 -2.336789000

O 0.448081000 2.611189000 -0.165422000

O -3.306315000 -2.325435000 0.406702000

O -1.359273000 -0.986018000 2.055530000

O 0.362060000 0.195060000 4.041037000

O -1.245968000 1.446384000 -1.843999000

O 1.246183000 -1.446479000 1.844179000

O -0.000180000 -0.000166000 -0.000274000

O 2.605127000 -0.460407000 -0.211140000

O 3.306297000 2.325398000 -0.406946000

O 1.359410000 0.986183000 -2.055708000

O 2.334329000 -3.324381000 -0.049931000

O -0.911374000 1.624757000 1.890021000

O -2.334247000 3.324514000 0.049525000

O -2.605261000 0.460461000 0.211131000

O -1.694020000 -1.165165000 -1.678758000

O 1.693903000 1.165190000 1.678792000

O -0.448153000 -2.611390000 0.165360000

O 0.911310000 -1.624591000 -1.889978000

W -1.911032000 -1.346272000 0.235816000

W -1.350743000 1.922156000 0.029283000

W 1.911024000 1.346241000 -0.236070000

W 1.350614000 -1.922197000 -0.029042000

W 0.209763000 0.111737000 2.336891000

O -0.361956000 -0.194986000 -4.040929000

**Table S10:** XYZ coordinates for **$[W_6O_{19}]^{3-}$**.

W -2.366125000 -0.101879000 0.157725000

O 0.113626000 1.726411000 2.038034000

O 0.188725000 -4.055405000 -0.354457000

O 1.953867000 -1.781499000 -0.287463000

O 4.065032000 0.175305000 -0.273284000

O -1.703042000 -0.239922000 2.050699000

O 1.753834000 0.235948000 -1.985775000

O -0.013253000 -0.000772000 0.000019000

O -0.149933000 2.040864000 -1.718920000

O -0.113776000 4.058712000 0.348256000

O -1.911623000 1.851658000 0.289451000

O -0.247798000 0.342308000 -4.052814000

O 2.016152000 -0.075011000 1.734397000

O 0.322226000 -0.338652000 4.047793000

O 0.254939000 -2.037490000 1.713735000

O -1.766287000 -2.010382000 -0.043192000

O 1.814703000 1.945013000 0.033922000

O -0.009329000 -1.722048000 -2.045620000

O -1.975962000 0.081527000 -1.804929000

W 0.078902000 -2.342299000 -0.203325000

W 0.156240000 -0.198698000 2.338521000

W -0.096944000 2.341609000 0.205160000

W -0.174527000 0.196839000 -2.337200000

W 2.346916000 0.102194000 -0.156814000

O -4.078374000 -0.175909000 0.272537000

**Table S11:** XYZ coordinates for **$[VW_5O_{19}]^{3-}$**.

V 0.000078000 0.001275000 -2.502779000

O -1.968309000 1.805386000 -0.191583000

O 0.169808000 -4.049604000 -0.244685000

O 0.083390000 -1.879256000 1.675319000

O -0.000334000 0.002181000 3.852216000

O -1.838464000 -0.083697000 -2.058421000

O 1.879987000 0.085833000 1.673435000

O -0.000212000 -0.000029000 -0.145259000

O 1.805472000 1.968026000 -0.191572000

O -0.169827000 4.049590000 -0.247735000

O -0.081586000 1.835911000 -2.059076000

O 4.049839000 0.169417000 -0.246012000

O -1.880291000 -0.081038000 1.673377000

O -4.049700000 -0.170170000 -0.246404000

O -1.805414000 -1.968179000 -0.186955000

O 0.081722000 -1.839274000 -2.056843000

O -0.083517000 1.882225000 1.671730000

O 1.968066000 -1.805271000 -0.186903000

O 1.838807000 0.079605000 -2.058354000

W 0.096971000 -2.326652000 -0.237632000

W -2.326775000 -0.097142000 -0.238262000

W -0.097056000 2.326659000 -0.236793000

W 2.326921000 0.096869000 -0.238127000

W -0.000054000 -0.000306000 2.125270000

O 0.000273000 -0.000036000 -4.094512000

**Table S12:** XYZ coordinates for **$[VW_5O_{19}]^{4-}$**.

V 0.000643000 -0.000478000 -2.553285000

O 2.646107000 -0.440212000 -0.146967000

O -2.368260000 3.308491000 -0.189022000

O -1.087350000 1.526599000 1.688104000

O -0.000050000 0.000163000 3.873454000

O 1.570183000 1.120129000 -2.042766000

O -1.526091000 -1.086790000 1.689069000

O 0.000351000 -0.000576000 -0.170180000

O -0.440310000 -2.645840000 -0.147526000

O 2.367500000 -3.308174000 -0.189221000

O 1.120373000 -1.569774000 -2.042703000

O -3.308016000 -2.368218000 -0.189247000

O 1.526145000 1.086772000 1.688967000

O 3.308580000 2.367591000 -0.188972000

O 0.440293000 2.646274000 -0.146876000

O -1.119506000 1.569490000 -2.042032000

O 1.086843000 -1.526642000 1.689565000

O -2.646539000 0.439936000 -0.147186000

O -1.569543000 -1.120207000 -2.042937000

W -1.359155000 1.897792000 -0.248050000

W 1.897620000 1.359053000 -0.248776000

W 1.358696000 -1.897394000 -0.248268000

W -1.897716000 -1.358683000 -0.248973000

W 0.000261000 -0.000533000 2.135847000

O 0.000164000 0.000181000 -4.164289000

**Table S13:** XYZ coordinates for **$[VW_5O_{18}(OH)]^{3-}$**.

| | | | |
|---|---|---|---|
| V | -0.244800 | -0.022400 | -2.581800 |
| O | 2.026200 | -1.720400 | -0.358600 |
| O | -0.269900 | 4.026900 | -0.244100 |
| O | 0.073400 | 1.869200 | 1.632400 |
| O | 0.257700 | 0.046500 | 3.860300 |
| O | 1.805500 | 0.070700 | -2.291700 |
| O | -1.748100 | -0.096700 | 1.845700 |
| O | 0.015400 | 0.002100 | -0.186000 |
| O | -1.753400 | -2.003600 | 0.020000 |
| O | 0.291200 | -4.025100 | -0.196900 |
| O | -0.037900 | -1.924100 | -2.055700 |
| O | -4.069400 | -0.284600 | 0.113900 |
| O | 2.064000 | 0.151400 | 1.585400 |
| O | 4.066700 | 0.270400 | -0.416100 |
| O | 1.769500 | 1.961500 | -0.413400 |
| O | -0.308300 | 1.883700 | -2.080300 |
| O | 0.311600 | -1.814500 | 1.663900 |
| O | -2.016400 | 1.747800 | -0.004000 |
| O | -2.104700 | -0.153800 | -1.903400 |
| W | -0.189600 | 2.302200 | -0.299400 |
| W | 2.353900 | 0.168200 | -0.249700 |
| W | 0.146100 | -2.305200 | -0.268900 |
| W | -2.361100 | -0.167900 | -0.094100 |
| W | 0.097600 | 0.023100 | 2.140100 |
| O | -0.369100 | -0.033800 | -4.177800 |
| H | 2.133500 | -0.774400 | -2.637600 |

## 9. References


(1) Yu, H.-Z.; Yang, Y.-M.; Zhang, L.; Dang, Z.-M.; Hu, G.-H. Quantum-Chemical Predictions of PK a's of Thiols in DMSO. *J. Phys. Chem. A* **2014**, *118*, 606–622.

(2) Bordwell, F. G.; Branca, J. C.; Bares, J. E.; Filler, R. Enhancement of the Equilibrium Acidities of Carbon Acids by Polyfluoroaryl Substituents. *J. Org. Chem.* **1988**, *53*, 780–782.

(3) Maran, F.; Celadon, D.; Severin, M. G.; Vianello, E. Electrochemical Determination of the PKa of Weak Acids in N,N-Dimethylformamide. *J. Am. Chem. Soc.* **1991**, *113*, 9320–9329.

(4) Kütt, A.; Tshepelevitsh, S.; Saame, J.; Lõkov, M.; Kaljurand, I.; Selberg, S.; Leito, I. Strengths of Acids in Acetonitrile. *Eur. J. Org. Chem.* **2021**, *2021*, 1407–1419.

(5) Tshepelevitsh, S.; Kütt, A.; Lõkov, M.; Kaljurand, I.; Saame, J.; Heering, A.; Plieger, P. G.; Vianello, R.; Leito, I. On the Basicity of Organic Bases in Different Media. *Eur. J. Org. Chem.* **2019**, *2019*, 6735–6748.

(6) Vallaro, M.; Ermondi, G.; Saame, J.; Leito, I.; Caron, G. Ionization and Lipophilicity in Nonpolar Media Mimicking the Cell Membrane Interior. *Bioorg. Med. Chem.* **2023**, *81*, 117203.

(7) Raamat, E.; Kaupmees, K.; Ovsjannikov, G.; Trummal, A.; Kütt, A.; Saame, J.; Koppel, I.; Kaljurand, I.; Lipping, L.; Rodima, T.; Pihl, V.; Koppel, I. A.; Leito, I. Acidities of Strong Neutral Brønsted Acids in Different Media. *J. Phys. Org. Chem.* **2013**, *26*, 162–170.

(8) Wise, C. F.; Agarwal, R. G.; Mayer, J. M. Determining Proton-Coupled Standard Potentials and X–H Bond Dissociation Free Energies in Nonaqueous Solvents Using Open-Circuit Potential Measurements. *J. Am. Chem. Soc.* **2020**, *142*, 10681–10691.

(9) Cooney, S. E.; Fertig, A. A.; Buisch, M. R.; Brennessel, W. W.; Matson, E. M. Coordination-Induced Bond Weakening of Water at the Surface of an Oxygen-Deficient Polyoxovanadate Cluster. *Chem. Sci.* **2022**, *13*, 12726–12737.

(10) Abraham, M. H.; Grellier, P. L.; Prior, D. V.; Duce, P. P.; Morris, J. J.; Taylor, P. J. Hydrogen Bonding. Part 7. A Scale of Solute Hydrogen-Bond Acidity Based on Log K Values for Complexation in Tetrachloromethane. *J. Chem. Soc., Perkin Trans. 2* **1989**, *0*, 699–711.
